# Rather doomed than uncertain: risk attitudes and transmissive behavior under asymptomatic infection

**DOI:** 10.1007/s00199-022-01448-y

**Published:** 2022-07-23

**Authors:** Konstantin Matthies, Flavio Toxvaerd

**Affiliations:** 1 Amazon Japan G.K., Tokyo, Japan; 2 Faculty of Economics, University of Cambridge, Austin Robinson Building, Sidgwick Avenue, Cambridge, CB3 9DD UK; 3 CEPR, London, UK

**Keywords:** Economic epidemiology, Risk aversion, Asymptomatic infection, Rational fatalism, COVID-19, I12, D81

## Abstract

We analyze the relation between individuals’ risk aversion and their willingness to expose themselves to infection when faced with an asymptomatic infectious disease. We show that in a high prevalence environment, increasing individuals’ risk aversion increases their propensity to engage in transmissive behavior. The reason for this result is that as risk aversion increases, exposure which leads to infection with certainty becomes relatively more attractive than the uncertain payoffs from protected behavior. We provide evidence from a laboratory experiment which is consistent with our theoretical findings.

**JEL Classification** I12 · D81

## 1 Introduction

The ongoing COVID-19 pandemic has emerged as one of the primary public health challenges of our time. COVID-19 is a respiratory disease that is transmitted primarily when people are in close physical proximity. Any activity that exposes people to others can pose a risk of infection. Accessing the public space or engaging in everyday

We thank Julien Gagnon, Edoardo Gallo, Sönje Reiche, Daniel Sgroi and Jakob Berndt for useful comments on this paper. We also thank the editor Nicholas Yannelis and three anonymous referees for very useful suggestions.

✉ Flavio Toxvaerd
fmot2@cam.ac.uk

1 Amazon Japan G.K., Tokyo, Japan

2 Faculty of Economics, University of Cambridge,
Austin Robinson Building, Sidgwick Avenue, Cambridge CB3 9DD, UK

3 CEPR, London, UK

social and economic activity such as shopping, attending school or going to work can therefore be considered risky. A particularly challenging feature of COVID-19 is that a large proportion of infected people show no symptoms. This makes it difficult for people not only to assess the infection status of people they meet, but also to accurately determine their own health status and thus their susceptibility to infection. Decision making in such an environment is therefore necessarily one of choosing between different uncertain prospects, or lotteries over different states of nature (i.e. health states). In addition, decision making is determined by people's underlying preferences over different outcomes and in particular, will depend on people's attitudes to risk.

How then, should one expect risk attitudes to influence people's exposure decisions during an epidemic? It is commonplace to see informal assertions that people who choose to expose themselves to infection risk are necessarily individuals that have high risk tolerance or are even risk-seeking. In other words, high risk aversion is often equated to strong incentives to self-protect. In this paper, we study the link between risk aversion and voluntary risk exposure under asymptomatic infection formally and experimentally. We show that in high prevalence environments where the probability of meeting infected people is high, increased risk aversion can in fact lead people to more, rather than less, exposure. This result holds under very general and standard assumptions on preferences and does not depend on specific functional forms. This result is further strengthened when protection is imperfect so individuals cannot fully eliminate exposure to infection.[1]

In our model, we assume that while transmissive behavior is intrinsically desirable (e.g. because protection is costly), it also carries the risk of infection. This is the basic tradeoff faced by the individual in deciding between protected and unprotected activity. A priori, one may think that a more risk averse individual would be less inclined to exposure and thus that, ceteris paribus, those individuals who engage in transmissive activity are likely to be relatively more risk tolerant. For an individual who is almost certainly healthy, this intuition turns out to be correct. This is because lack of exposure yields a known constant payoff to the healthy individual (when protection confers perfect immunity), while unprotected exposure leads to uncertainty over the individual's final health status. As risk aversion increases, protected behavior becomes even more attractive to the individual, *relative* to unprotected behavior.

The problem with extrapolating from this line of reasoning is that it ignores the possibility that the individual is already (asymptomatically) infected and once this possibility is factored in, the result can be reversed.[2] To better understand the intuition for this finding, consider the stark case in which unprotected exposure leads to infection with certainty. In this case, the expected payoff from exposure (i.e. the positive benefits from engaging in unrestricted social activity) is constant and known, while protected behavior leads to an uncertain outcome because the basic uncertainty over the individual's initial health status remains unresolved. For that reason, a more risk averse individual would value unprotected exposure relatively higher than a less risk averse individual. This is not to say that the risk averse individual *wants* to become

[1] This result is linked to *rational fatalism*, which is said to occur when an individual's response to increases in prevalence lead to an increase in exposure.

[2] Delavande and Kohler (2009) and De Paula et al. (2014) study how beliefs about HIV infection status can influence people's sexual decision making.

infected. Rather, *relative to a more risk tolerant individual*, the individual values the relative certainty over outcomes resulting from unprotected exposure to infection more than the uncertainty resulting from protected behavior.

We formalize these counter-intuitive results and prove that under perfect protection, an increase in risk aversion changes the tradeoffs in such a manner that individuals facing low prevalence environments become more inclined towards protected behavior, while individuals facing high prevalence environments become more inclined towards exposing themselves to infection. An example of such an environment includes a household where one member is already known to be infected and where it is difficult to engage in social distancing. We show via examples that our results hold for standard utility functions and empirically plausible levels of risk aversion. Thus, although our results may at first seem surprising, they in fact follow from perfectly standard assumptions.

Next, we consider the effects of imperfect protection and show that it leads to disease exposure becoming even more attractive for more risk averse individuals facing high risk environments. The reason for this is that imperfections in protection increase the uncertainty over outcomes resulting from protected exposure, while not influencing those resulting from unprotected exposure. We show that when protection is sufficiently imperfect, increasing risk aversion unambiguously increases the propensity to expose oneself, irrespective of the disease prevalence in the environment.

In addition to the theoretical analysis, we also empirically test our model. We conduct a pilot laboratory experiment to gather empirical evidence, which provides valuable insights for the design of field studies. We use traditional risk elicitation tasks to control for subjects' risk attitudes and estimate the impact of these attitudes on behavior in a setting that mirrors our model. We find some evidence that increased risk aversion leads to increased risk exposure choices in high risk environments, in line with our model's predictions.

While the literature on decision making and infectious diseases is both voluminous and varied, only a small number of papers are closely related to our setup.[3] Philipson and Posner (1993) were the first to formalize the tradeoffs involved in decision making when individuals are uncertain about their health status and that of their partner(s). They show that for an individual who is sufficiently likely to already be infected, the privately optimal course of action may indeed be to engage in unprotected behavior. Under certain conditions, uncertainty about one's own health status can lead to so-called rational fatalism, i.e. the phenomenon that an increase in the perceived riskiness of unprotected activity leads to an increase in the propensity to engage in unprotected behavior. Rational fatalism is a central finding in Kremer (1996) and Auld (2003) in models of partner change. For our purposes, it is important to note that rational fatalism in deciding on the marginal exposure to infection is not intrinsically related to risk attitudes, but rather to uncertainty about one's own health status. Indeed, while Kremer (1996) and Auld (2003) implicitly assume that individuals are risk averse, Philipson and Posner (1993) confine attention to risk neutral individuals. As long as the perceived riskiness of unprotected behavior is allowed to affect an individual's beliefs about their

[3] Contributions such as Makris (2021) assume perfect health state information and risk neutrality and focus on other aspects of decision making.

own health status, rational fatalism can occur. More recent contributions on different aspects of belief formation and fatalism include Sterck (2012, 2014) and Kerwin (2012, 2020). We will discuss these contributions further below. We should also note that this notion of fatalism is intrinsically different from that studied by Shapiro and Wu (2011).

Last, we should note papers by Greenwood et al. (2019) and Toxvaerd (2017). These papers extend the problem of asymptomatic infection and decision making to dynamic contexts, where learning about health states becomes a central issue. These papers do not primarily concern themselves with the effects of risk aversion.

In Sect. 2, we present a model of risky behavior with asymptomatic infection. In Sect. 3, we analyze the effects of risk aversion on the decision to engage in unprotected exposure. In Sect. 4, we consider the effects of imperfect protection. In Sect. 5, we report on evidence from a laboratory experiment. Section 6 concludes. Proofs of all main results are relegated to the “Appendix”, as is a detailed exposition of our experimental analysis.

## 2 The model

Consider an individual who is contemplating engaging in some potentially transmissive behavior or activity. The individual can choose an action $a \in \{R, S\}$, where $a = R$ denotes the option that involves exposure, like accessing the public space, and $a = S$ denotes choosing a safe alternative, like staying home.[4] Individuals can be in one of two health states, namely healthy or infected. We denote the health state of the individual by $h \in \{H, I\}$, where $h = H$ denotes healthy and $h = I$ denotes infected. The infection is assumed to be asymptomatic, so the individual does not directly observe their health state. Instead, we assume that the individual holds a subjective prior belief $p \in [0, 1]$ that they are already infected. In addition, we assume that individuals form beliefs about the risks involved in exposing themselves to infection. Such beliefs can be derived from knowledge of aggregate disease prevalence or based on public announcements from health authorities. Turning to preferences, we assume that the individual is endowed with a strictly increasing and concave utility function $u\left(x_h^a\right)$, where $x_h^a$ denotes one of four possible final outcomes. The interpretation of $x_h^a$ is that it is a composite bundle that captures both the individual's ex-post health state and any cost of risk mitigation, i.e. the final outcome will depend on the health state and the action chosen by the individual. We assume that

$$x_H^R > x_H^S > x_I^R > x_I^S \quad (1)$$

These inequalities mean that while both infection and protection are costly, the cost of infection outweighs the cost of protection. In particular, this implies that if there is no risk of infection transmission, then the individual will prefer to expose themself fully and avoid the cost of mitigation. Furthermore, a healthy individual would rather pay

[4] For mnemonic reasons, we use $R$ and $S$ to denote the *risky* and *safe* option, respectively. As will become clear in what follows, the terms *transmissive* and *non-transmissive* behavior are perhaps more appropriate, but our use of language should not cause any misunderstanding.

the price of protection, than bear the cost of infection with certainty. A special case of these preferences over final outcomes are those where utility is separable in health states and protection choices. With such preferences, the individual incurs some cost $c > 0$ for choosing to protect against infection and experiences some disutility $D > 0$ if they end up infected.

Assume that the individual assigns probability $q \in [0, 1]$ to being exposed to infected individuals and probability $\beta \in [0, 1]$ to the infection being transmitted during such exposure, conditional on the person being healthy. Were the individual healthy, they would therefore assign probability $r \equiv \beta q$ to becoming infected as a result of unprotected exposure. We will refer to $r \in [0, 1]$ as the *risk* of infection.

The probability assigned to the individual ending up in the infected state depends both on the prior belief, on the individual's decision and on the risk they face. For ease of notation, define

$$p^S \equiv p, \quad p^R \equiv p + (1 - p)\, r \tag{2}$$

These are simply the ex post probabilities of being infected under protected and unprotected behavior, respectively.[5] In words, under the safe choice, the probability that the individual will end up being infected is simply the probability that the individual was infected at the outset. Under the risky choice, there is the additional possibility of becoming infected through the exposure.

With this notation in place, the expected utility of an individual who chooses exposure is given by

$$U^R \equiv \left(1 - p^R\right) u\left(x_H^R\right) + p^R u\left(x_I^R\right) \tag{3}$$

Similarly, the expected utility from the safe behavior is given by

$$U^S \equiv \left(1 - p^S\right) u\left(x_H^S\right) + p^S u\left(x_I^S\right) \tag{4}$$

The individual's optimal decision depends on the relative magnitudes of $U^R$ and $U^S$. Indifference between unprotected and protected behavior obtains for combinations $(r^*, p^*)$ for which $U^R = U^S$, or

$$\begin{aligned}\left(1 - p^*\right) u\left(x_H^S\right) + p^* u\left(x_I^S\right) &= \left(1 - \left[p^* + \left(1 - p^*\right) r^*\right]\right) u\left(x_H^R\right) \\ &+ \left[p^* + \left(1 - p^*\right) r^*\right] u\left(x_I^R\right)\end{aligned} \tag{5}$$

In $(r, p)$-space, this gives rise to a *switching curve* given by the function

$$p^*(r) \equiv \frac{(1 - r)\, u\left(x_H^R\right) - \left[u\left(x_H^S\right) - u\left(x_I^R\right)\right]}{(1 - r)\left[u\left(x_H^R\right) - u\left(x_I^R\right)\right] - \left[u\left(x_H^S\right) - u\left(x_I^S\right)\right]} \tag{6}$$

[5] Note that in this formulation, protection is perfect and reduces the transmission probability to zero. Later, we will relax this assumption.

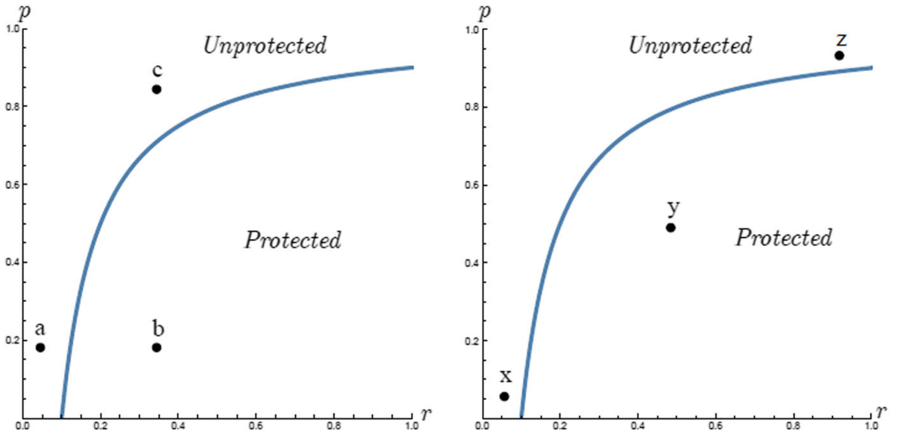

**Fig. 1** Switching curves in $(r, p)$-space, increasing risks and fatalism

Given a transmission risk $r$, for $p > p^*(r)$ the individual prefers unprotected exposure, while for $p < p^*(r)$, the individual prefers the safe option. Thus the higher the individual's subjective assessment of already being infected is, the more willing will they be to choose the risky option. An example of a switching curve in $(r, p)$-space, based on the constant relative risk aversion (CRRA) utility function, is shown in Fig. 1. Note that for combinations $(r, p)$ below the switching curves, the individual prefers the safe option, while for combinations above it, the individual prefers the risky option.

To better appreciate the basic working of the model, consider the left-hand side panel and compare the points $a$, $b$ and $c$. A movement from point $a$ to point $b$ constitutes an increase in the risk associated with exposure and will, ceteris paribus, cause the individual to switch from preferring the risky option to preferring the safe option. In contrast, a switch from point $b$ to point $c$ constitutes an increase in the probability that the individual is already infected and will, ceteris paribus, cause the individual to switch from preferring the safe option to preferring the risky option.

In the right-hand side panel, we illustrate the phenomenon known as rational fatalism. Consider simultaneous increases in both the individual's infection probability and in the risk facing the individual, e.g. like the movement between the points $x$, $y$ and $z$ that all lie on the 45°-line. Because the switching curve is concave in $(r, p)$-space, the 45°-line can intersect the switching curve twice. A switch from point $x$ to point $y$ will cause a switch from risky to safe behavior. But a switch from point $y$ to point $z$ will cause a switch from safe to risky behavior. This is exactly the phenomenon known as rational fatalism, i.e. the tendency to respond to a perceived increase in 'risk' by increasing supposedly 'risky behavior'.[6] The different contributions to the literature give different accounts for how a shift like the one from point $y$ to point $z$ can occur. In Philipson and Posner (1993), individuals' infection probabilities are drawn from the same distribution and when the distribution shifts in the sense of first-order stochastic

[6] Non-monotonicities in disease risks are also found in the testing literature, like Boozer and Philipson (2000). But these are driven by value of information considerations that are somewhat different than the ones considered here.

dominance, both the individual's infection probability and overall disease prevalence increases. In Kremer (1996), individuals commit to a number of exposures. When the overall infection risk increases, either because of increases in aggregate prevalence or because of increases in the transmission probability, the individual 'is moved' from a point like $y$ to a point like $z$. A similar effect is at work in the model of Kerwin (2012, 2020). In Kerwin (2020), a decrease in the perceived infectiousness of HIV can simultaneously shift an individual's beliefs about the risks from additional sexual encounters downwards and increase the individual's probability assessment that they have not previously been infected and is thus still at risk. This can induce fatalistic responses to changes in information. Kerwin (2020) provides interesting empirical evidence of such responses in the context of sexual decision making in Malawi. The simultaneous updating of beliefs about external risks and personal susceptibility is also prominent in Toxvaerd (2017), in the context of repeated monogamous interactions.

Last, in the context of within-partnership transmission, a long-term partner's health status may be highly correlated with that of the individual, as shown by Toxvaerd (2017). In this case, once the partner shows symptoms of being infected, the individual may find it more likely to be infected themself, thus prompting a switch to less prevention.

## 3 Risk aversion and transmissive behavior

We will now take a closer look at how the individual's attitudes to risk determine their decisions vis-à-vis transmissive behavior. From inspection of the switching curve (5), it is trivially true that the decision on exposure versus protection depends on the shape of the utility function, to wit on the individual's risk attitudes.

To trace the effects of varying risk aversion on the individual's decision, we will next determine how the individual's switching curve moves in $(r, p)$ -space as the coefficient of local risk aversion $\theta(x)$ changes. For concreteness, one can think of $\theta(x)$ as being either the coefficient of absolute risk aversion $A(x) \equiv -u''(x)/u'(x)$ or the coefficient of relative risk aversion $R(x) \equiv -xu''(x)/u'(x)$, but our results do not depend on these particular representations.

To trace the shifts in the switching curves, it is useful to consider the extremes in $(r, p)$-space. We start by considering what happens as $p \to 0$, i.e. when the individual is virtually certain that they are healthy to start with. Let $r_i^*(p)$ denote the value of $r$ that makes the individual indifferent between unprotected and protected behavior under prior belief $p$ and risk aversion coefficient $\theta_i(x)$. We then have the following result:

**Proposition 1** *Let $u(x)$ be a well-behaved utility function with local risk aversion coefficient $\theta(x)$. For any $\theta_2(x) > \theta_1(x)\, \forall x$, it is the case that $\lim_{p\to 0} r_2^*(p) < \lim_{p\to 0} r_1^*(p)$.*

This result means that for an individual who is almost certainly susceptible, as the coefficient of risk aversion increases, the individual's willingness to self-protect increases. This results chimes with the common intuition outlined in the introduction. It follows from the observation that for an uninfected individual, the safe option provides a known payoff with certainty (since protection is assumed to be perfect). In contrast, the risky option yields an uncertain payoff, because there is uncertainty over whether the individual will become infected from unprotected exposure. Thus, in this setting, increasing risk aversion unambiguously makes the safe option relatively more attractive. Let $p_i^*(r)$ denote the critical value given by (6) for an individual with risk aversion coefficient $\theta_i(x)$. An immediate corollary of Proposition 1 is as follows:

**Corollary 2** *Let $u(x)$ be a well-behaved utility function with local risk aversion coefficient $\theta(x)$. For any $\theta_2(x) > \theta_1(x)\,\forall x$, there exists a range of transmission risks $r$ such that $p_2^*(r) > p_1^*(r)$.*

To understand this result, note that Proposition 1 implies that the switching curve $p_2^*(r)$ of a more risk averse individual will intersect the $r$-axis for a smaller value of $r$ than the switching curve $p_1^*(r)$. Continuity of equation (6) then implies that there must be a least some range of risks $r$ for which $p_2^*(r)$ lies above $p_1^*(r)$.

Next, we consider another extreme, namely the case where $r \to 1$. In this case, an unprotected encounter will result in the individual ending up infected with certainty (either as a result of becoming infected though the unprotected activity or because the individual was already infected at the outset). Our result for this case is a follows:

**Proposition 3** *Let $u(x)$ be a well-behaved utility function with local risk aversion coefficient $\theta(x)$. For any $\theta_2(x) > \theta_1(x)\,\forall x$, it is the case that $\lim_{r\to 1} p_2^*(r) \leq \lim_{r\to 1} p_1^*(r)$.*

This result may seem counter-intuitive at first blush. It states that as infection from unprotected exposure becomes almost certain, an increasingly risk averse individual becomes more inclined to engage in the unprotected activity! To understand this result, recall our discussion above, comparing uncertain prospects. For an individual that engages in unprotected behavior, the outcome is known with certainty when $r \to 1$. That is, the individual knows that in this case, if they engage in transmissive behavior, they will end up infected (irrespective of the individual's initial infection status). On the other hand, the safe option (which we have for now assumed leads to perfect protection against any new infection) still leaves the individual with uncertain prospects. In particular, the individual still assigns the initial probability $p$ to being infected from the outset and probability $(1 - p)$ to being healthy. Under the safe option, this uncertainty remains. The upshot of this is that unprotected exposure now provides the individual with a known payoff for sure, whereas engaging in protected behavior means that the individual's prospects remain uncertain. [7] As the individual becomes increasingly risk averse, the uncertainty associated with the safe option becomes increasingly unpalatable, with the individual therefore opting more readily for unprotected behavior.

An immediate corollary of Proposition 3 is as follows:

**Corollary 4** *Let $u(x)$ be a well-behaved utility function with local risk aversion coefficient $\theta(x)$. For any $\theta_2(x) > \theta_1(x)\,\forall x$, there exists a range of transmission risks $r$ such that $p_2^*(r) < p_1^*(r)$.*

7 This is exactly the sense in which the terms *risky* and *safe* behavior can be misleading.

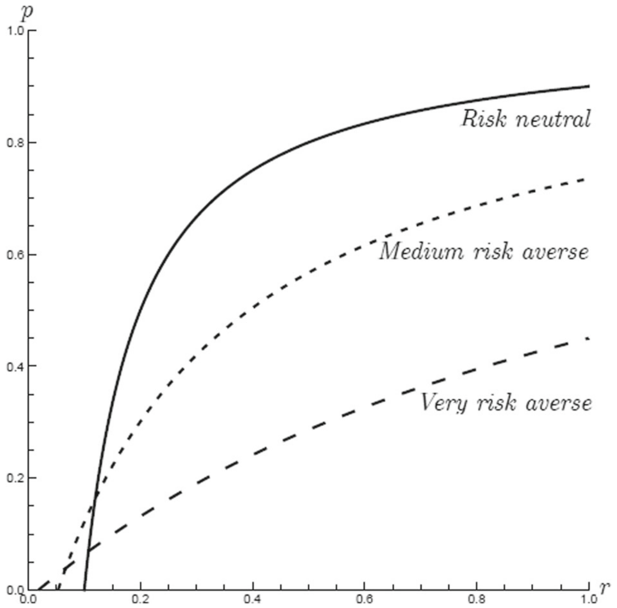

**Fig. 2** Switching curves in $(r, p)$-space for different degrees of risk aversion

The rationale for this result is similar to that of the previous Corollary. Figure 2 shows the shifts in the switching curves for different levels of risk aversion. As can be seen from the figure, an increase in risk aversion causes the switching curve to pivot clockwise, thereby conforming to the characterization given in the previous propositions.

It is important to emphasize that our findings do not rely on the assumption that individuals have unrealistically high levels of risk aversion. To see this clearly, it is instructive to consider an explicit numerical example. Suppose that the individual has constant relative risk aversion and that payoffs are given by $(x_H^R, x_H^S, x_I^R, x_I^S) = (6, 5.5, 0.5, 0)$. In their experimental study, Holt and Laury (2002) estimate that at least 80% of their subjects have coefficients of relative risk aversion $\theta$ for CRRA preferences in the range $[0, 0.9]$. As the coefficient of relative risk aversion $\theta$ increases from 0 to 0.9, the critical value determining the switch from risky to safe behavior at risk $r = 1$ decreases from a value around 90% to around 21%. In other words, for individuals in a high prevalence environment, a reasonable variability in risk aversion can create quite dramatic changes in their willingness to engage in unprotected behavior. This result is quite robust, in that it continues to hold even when the stakes are higher. Consider the alternative example $(x_H^R, x_H^S, x_I^R, x_I^S) = (100, 90, 10, 0)$, where we have amplified the shortfall in utility experienced by an infected individual. In this case, as the coefficient of relative risk aversion $\theta$ increases from 0 to 0.9, the critical value determining the switch from risky to safe behavior decreases from a value around 88% to around 20%. In fact, the dramatic fall happens even for an individual that faces less extreme risks. In the high-stakes example, the shift for an individual facing a more moderate risk, say 50%, would experience a drop from 78% to only 10%. Similar drops will be present for even lower risks, as long as we consider points to the right of the intersection of the switching curves, which can in turn be located very close to the origin. What these examples show is that there is nothing extreme or pathological about our setup. The results hold for reasonable preferences and for empirically plausible values of risk aversion.

## 4 The effects of imperfect protection

In the previous section, we made the strong assumption that protection perfectly shields the individual from additional infection risks. In this section, we relax this assumption and allow for the possibility that the protected option does not confer perfect immunity. As will become clear, this possibility strengthens our main message.

In particular, we assume that protection is subject to a failure probability $\phi \in [0, 1]$. To fix ideas, one can think of $\phi$ as the probability of unavoidable contacts with others, such as with family members or carers. While imperfect protection does not alter the expected utility from the unprotected option $U^R$, the expected utility from the safe option changes to

$$\hat{U}^S \equiv \left(1 - p^S\right)\left[(1-\phi)\, u\left(x_H^S\right) + \phi\left[(1-r)\, u\left(x_H^S\right) + ru\left(x_I^S\right)\right]\right] + p^S u\left(x_I^S\right) \tag{7}$$

To understand this expression, note that with probability $(1 - p^S)$, the individual is susceptible to infection. Under the safe option, with probability $(1-\phi)$ the protection works as intended and the individual remains healthy. But with probability $\phi$ there is a failure in protection, thus exposing the individual to the risk of infection. Whether this risk materializes depends on the transmission risk $r$, exactly as is the case for the risky option.

The switching curve in this setting is given by the combinations $(r, p)$ such that $U^R = \hat{U}^S$. Solving for the prior $p$, this gives the modified function

$$p^*(r, \phi) \equiv \frac{(1-r)\, u\left(x_H^R\right) - \left[\left[(1-\phi r)\, u\left(x_H^S\right) + \phi r u\left(x_I^S\right)\right] - u\left(x_I^R\right)\right]}{(1-r)\left[u\left(x_H^R\right) - u\left(x_I^R\right)\right] - \left[\left[(1-\phi r)\, u\left(x_H^S\right) + \phi r u\left(x_I^S\right)\right] - u\left(x_I^S\right)\right]} \tag{8}$$

Our first result in this setting is as follows:

**Proposition 5** *Let $u(x)$ be a well-behaved utility function with local risk aversion coefficient $\theta(x)$. For any $\theta_2(x) > \theta_1(x)\, \forall x$, it is the case that* $\lim_{r\to 1} p_2^*(r, \phi) < \lim_{r\to 1} p_1^*(r, \phi)$.

This result is equivalent to that under perfect protection, with the levels adjusted for the possibility of protection failure. When infection is the inevitable consequence of unprotected behavior, then this behavior yields a known future payoff with certainty. In contrast, the safe option still involves some uncertainty, because the individual has prior probability $p$ of already being asymptomatically infected.

It is more interesting to consider what happens away from the limit, i.e. when $r < 1$. In this case, there is a proper tradeoff in terms of uncertainty, because both risky and safe behavior offer uncertain prospects. It turns out that this tradeoff is nicely parameterized by the failure probability $\phi$. Formally, we have the following result:

**Proposition 6** *Let $u(x)$ be a well-behaved utility function with local risk aversion coefficient $\theta(x)$. For any $\theta_2(x) > \theta_1(x)\,\forall x$, there exists a threshold value $\bar{\phi}$ such that for $\phi \leq \bar{\phi}$, $\lim_{p\to 0} r_2^*(p,\phi) \leq \lim_{p\to 0} r_1^*(p,\phi)$, while for $\phi > \bar{\phi}$, $\lim_{p\to 0} r_2^*(p,\phi) > \lim_{p\to 0} r_1^*(p,\phi)$.*

This result allows us to make a number of interesting observations. First, when the failure probability is sufficiently low (but not necessarily zero), the qualitative features of the perfect protection case continue to hold. Second, when the failure probability is sufficiently high, then we can totally order the individual's inclination to engage in protection by their coefficient of risk aversion. In particular, controlling for the prior $p$, we have the following:

**Proposition 7** *Under imperfect protection with sufficiently high failure probability, the higher the individual's coefficient of risk aversion $\theta(x)$ becomes, the less will the individual wish to engage in protection.*

***Proof*** Let $\mathcal{R}_i$ denote the upper contour set of the switching curve in $(r, p)$-space under risk aversion coefficient $\theta_i(x)$, $i = 1, 2$, with $\theta_2(x) > \theta_1(x)\forall x$. This means that if $(r, p) \in \mathcal{R}_i$, then an individual with risk aversion coefficient $\theta_i(x)$ would choose unprotected behavior. For sufficiently imperfect protection, the switching curves do not intersect and thus for $\phi > \bar{\phi}$, $\mathcal{R}_1 \subset \mathcal{R}_2$. This means that $(r, p) \in \mathcal{R}_1 \Rightarrow (r, p) \in \mathcal{R}_2$ and so the higher the level of risk aversion, the higher the propensity to engage in transmissive behavior, *irrespective* of prior beliefs $p$ □

Figure 3 shows the shifts in the switching curves and the critical values for different levels of risk aversion, for low and high failure probability, respectively.

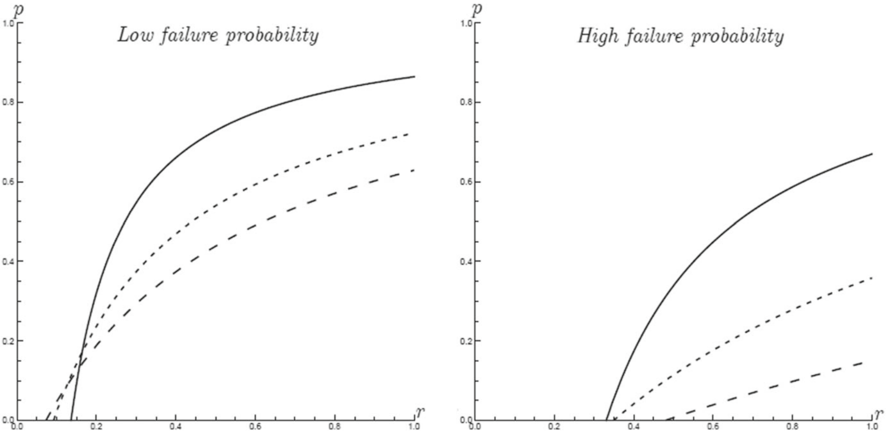

**Fig. 3** Switching curves in $(r, p)$-space for different degrees of risk aversion and imperfect protection. Left-hand side panel shows low failure probability; right-hand side panel shows high failure probability

As we have just noted, for $\phi \leq \bar{\phi}$ set inclusion only holds over some ranges of $r$, because the switching curves for different risk attitudes intersect. A weaker order than set inclusion is to compare the measures of the risky sets $\mu(\mathcal{R}_1)$ and $\mu(\mathcal{R}_2)$, but we have been unable to prove any ranking analytically.

## 5 Experimental evidence

To assess the external validity of our model, we test it empirically by developing a laboratory experiment that replicates the environment of our model. Ultimately, the theoretical predictions should be tested in the field, either through large population studies or field experiments. However, laboratory experiments can offer good initial stress tests of the theory since the laboratory environment allows complete freedom to alter the model's parameters. This allows direct testability of the above predictions, which are strongest in limit cases, that field experiments often do not.

While field experiments are a well-established practice in economic epidemiology, surprisingly little research has utilized laboratory experiments. Chapman et al. (2012) and Ibuka et al. (2014) investigate vaccination behavior in the lab, while Chen et al. (2013) are able to replicate behavior observed in real world epidemics in an online multi-player game. Sen (2004), whose work is closely related to ours, shows that changes in risk perception induced by HIV testing can result in increases in risky sexual behavior, driven partially by fatalism.

To better understand actual behavior and its relation to risk attitudes, we develop an experimental task that mirrors the theoretical model described in Sect. 2 and also collect several measures of risk aversion, including the Investment Game (Gneezy and Potters 1997; Charness and Gneezy 2010) and the Multiple Price List (Holt and Laury 2002), which we use to predict the effects of risk aversion on behavior in our model.

The experimental analysis provides some support to the core theoretical propositions. The Investment Game measure of risk aversion accurately predicts implied risk attitudes in the main task and verifies the theoretical prediction that risk aversion increases the propensity to act in a fatalistic manner. In a high-prevalence environment, we find that with increased risk aversion, subjects more readily switch to unprotected behavior. We also show that risk aversion increases overall levels of protection. Below, we briefly summarize the experimental design and the key findings. A detailed description of findings and additional treatments investigating framing effects can be found in "Appendix F".

### 5.1 Experimental design

Our experimental design consists of four parts. The first part replicates our model. The three following parts are three separate risk elicitation tasks performed in random order to control for potential learning effects. In addition, subjects complete a demographic questionnaire.[8]

[8] All parts and questionnaires are programmed in z-Tree (Fischbacher 2007).

### 5.1.1 Part 1: main task

Subjects are told they are contestants in a game show. We choose this "neutral" context for the experiment on purpose due to concerns about framing effects when discussing diseases (more on this in "Appendix F"). There are two consecutive rounds to the game show, "Round 1" and "Round 2", respectively. The aim of the game is to successfully hold on to the initial prize money of £6 for two rounds, in each of which there is a risk of losing the game. In case the subject loses in either round, £5.5 would be deducted from the earnings. The outcome of Round 1, however, is not revealed before Round 2 is played, so subjects are not aware whether they have already lost going into Round 2. Subjects have the option of not participating in and hence eliminating, Round 2 after Round 1 has been played. For simplicity we refer to eliminating Round 2 as "protecting" and playing Round 2 as "not protecting". Protecting costs the subject £0.5 and in this case, only the outcome of Round 1 will determine their winnings.

Subjects are taken through 192 scenarios, each uniquely identified by the risk $r$ of losing in Round 2 (16 different probabilities: 0%, 1%, 5%, 10%, 15%, 20%, 25%, 30%, 35%, 40%, 50%, 60%, 70%, 80%, 90%, 100%) and the likelihood of already having lost in Round 1, the prior $p$ (12 different probabilities: 0%, 5%, 10%, 20%,..., 90%, 100%). These scenarios are presented across four consecutive screens (see Fig. 6 in "Appendix H"), using tables displaying 4 different values for $r$ each and the full range of possible values for $p$. Each scenario refers to an entry in the table. Subjects are informed that both probabilities are independent and scenarios are evaluated separately and independently. For each scenario, they are then asked to choose whether to play Round 2 or not. They indicate their choice by ticking respective boxes in the table entry.

### 5.1.2 Parts 2–4: risk elicitation tasks

In this part subjects complete three separate risk elicitation tasks in random order.[9] Traditionally the literature has found little or no correlation between risk preferences elicited using different methods Lonnqvist et al. (2014). In particular, it has been shown that risk preferences can vary vastly across context (Dohmen et al. 2005, 2011; Szrek et al. 2012). As a result, we include several measures in our experiment while keeping the number manageable for subjects.

The tasks were selected from the existing literature and chosen according to a variety of criteria. We use the criteria described in Charness et al. (2013), such as relative simplicity and accuracy. We also consulted Crosetto and Filippin (2013a) who provide a detailed appraisal of five risk elicitation tasks and offer a theoretical comparison. We selected the "Investment Game: Charness, Gneezy and Potters (IG)" (Gneezy and Potters 1997 and Charness and Gneezy 2010) and the "Multiple Price List: Holt and Laury Version (MPL)" (Holt and Laury 2002).[10] Both measures are common in the literature and have advantages and disadvantages associated with them. While

9 Note that for logistical reasons, such as reading of the instructions, the order was identical for all subjects within one session.

10 Our MPL task is a heavily modified version of code used in Prasad and Salmon (2013).

the MPL is more commonly used in the literature, it is also more complex to understand and only allows for segmentation into 9 risk aversion ranges. The IG is considerably simpler and allows for an almost continuous classification of risk aversion. It does not, however, capture risk loving behavior. Furthermore, we include Crosetto and Filippin's own "Bomb Risk Elicitation Task (BRET)"(Crosetto and Filippin 2013b).[11] This task is included to provide a visual risk elicitation task as well as two strictly numerical ones.

#### 5.1.3 Investment game

Subjects are endowed with £2.00 and offered the chance to place any amount (in increments of £0.01) of their endowment in a 50/50 bet. With a 50% chance they win and earn 2.5 times their wager. With a 50% chance they lose their entire wager.[12] The expected payoff for this task is increasing in the amount wagered. Therefore a risk neutral individual should place the maximum £2.00. The lower the amount placed, the higher the degree of risk aversion. Subjects are allowed to test different amounts before making their final decision.

#### 5.1.4 Multiple price list

Subjects are presented with ten lottery pairs with a relatively safe lottery **A** and a relatively risky lottery **B**. Lottery **A** pays £2.00 upon winning and £1.60 upon losing. Lottery **B** pays £3.85 and £0.10 respectively.[13] Both lotteries have the same chances of winning or losing. The chance of winning starts at 10% in lottery pair 1 and increases by 10% for every pair. Lottery pair 10 therefore has a 100% chance of winning. For each pair, subjects are asked to indicate their preference for lottery **A** or **B**. A rational agent should always prefer lottery **B** in pair 10. As the chance of winning decreases to pair 1 they will switch at some point along the lottery pairs (or not for highly risk loving individuals) to the safer lottery **A**. The switching point is used to pinpoint the agent's risk preference.

In our experiment subjects have to express a strict preference between lotteries. We do not, however, restrict subjects to one switching point. Therefore, behavior inconsistent with rationality in the expected utility sense is allowed. We find that 96% of subjects display consistent behavior. Crosetto and Filippin (2013a, b) highlight that this task can be perceived as relatively complex. In order to help with the understanding, subjects can evaluate examples for lotteries.

#### 5.1.5 The bomb risk elicitation task

Subjects face a ten by ten field that can be filled with up to 100 blocks. Subjects start with zero blocks and as the game progresses, they automatically collect one block

[11] Our BRET is based on the code supplied by Paolo Crosetto on his website "https://paolocrosetto.wordpress.com/research/bret/" with some visual and functional modifications.

[12] Payoffs were chosen in accordance with Crosetto and Filippin (2013a, b).

[13] Payoffs were chosen in accordance with the literature, in particular Holt and Laury (2002) and Crosetto and Filippin (2013a, b).

every second. For every block they collect, they potentially earn £0.10. However, subjects are told that there is a bomb hidden behind one of the blocks that will destroy all their earnings should it be collected. The bomb hides behind any block with equal probability. The subjects' task is to decide when to stop collecting blocks by pressing a "STOP" button. This task involves a direct tradeoff between the money that can be earned and the likelihood of winning this money. A risk neutral individuals should choose to collect 50 boxes to maximize the expected payoff of the task. In order to help with the understanding of the task, subjects play one practice round of the task before having to make their recorded choice.

#### 5.1.6 Additional control variables

Subjects complete a demographic questionnaire that asks them to give their age, gender, years they have spent at university and their degree qualification. The questionnaire also includes a measure for fatalism as proposed in Shapiro and Wu (2011). This measure is taken from the 1992 National Longitudinal Survey of Youth. Subjects are asked whether they feel they "have little control over things that happen to me" (them). They choose on a 4 point scale to "Strongly Disagree", "Somewhat Disagree", "Somewhat Agree" or "Strongly Agree". With rational fatalism being a key implication of our theory, we believe testing for fatalism directly is important. We use this measure in addition to the traditional risk aversion measures.

#### 5.1.7 Payment

All payment in the experiment are expressed in points where 1 point = £0.05.[14] Payments and outcomes of individual tasks are not revealed until the end of the session to avoid house money effects. Subjects are paid a £5 show-up fee. Parts 1–4 were incentivized according to performance where outcomes are generated randomly by the computer given subjects' choices. In part 1 subjects receive payment for one randomly chosen scenario out of the environment that displayed odds and one randomly chosen scenario out of the environment that did not. In Parts 2-4 subjects receive payment for one randomly chosen lottery in the MPL and their full earnings in the IG and BRET. The average payout across sessions was approximately £17 and payments ranged from £6 up to £27.50 out of a theoretical maximum of £36.75.

### 5.2 Formal hypotheses

Collecting subjects' choices in the main task allows us to elicit implied switching curves. The propositions outlined in Sect. 3 indicate that an increase in risk aversion, denoted by $\theta$, will shift switching curves in the following ways:

1. If $\theta$ increases, then the switching curve's intercept with $p = 0$ will shift to the left.
2. If $\theta$ increases, then the switching curve's intercept with $r = 1$ will shift down.

[14] One exception was the Investment Game where payment was expressed in pence.

The first result implies that if an individual is unlikely to have lost in Round 1, then their propensity to protect will increase with risk aversion. This means that protection becomes optimal for lower levels of the risk of losing in Round 2, $r$. We denote the intercept with the $p = 0$ line as the *lower intercept*. An increase in risk aversion shifts the lower intercept to the left. We also seek to test the counter-intuitive result discussed in Sect. 3, which says that if losing is almost certain, then the propensity to protect will decrease with risk aversion. We denote the intercept of the switching curve with the $r = 1$ line as the *upper intercept*. An increase in risk aversion shifts the upper intercept downwards.

It is important to note that our theory predicts changes in the lower intercept to be relatively small, compared to changes in the upper intercept. For example, consider the case in which the payoffs from our experiment for $x_h^a$, preferences are CRRA

$$u\left(x_h^a\right) = \frac{x^{1-\theta}}{1-\theta} \tag{9}$$

and the distribution of risk aversion coefficients $\theta$ is that estimated in Holt and Laury (2002), namely $\theta \in [-1, 1.4]$ . An increase in risk aversion from the bottom of the distribution of $\theta$ to the top would imply a shift of the lower intercept from ca. 15% to 2% risk of losing in Round 2. In contrast, for the upper intercept it implies a shift from ca. 90% to 35%. This means that changes in the lower intercept might be harder to detect in the data. Note further that our analysis does not actually require estimation of $\theta$ as we can use the direct choices observed in the risk elicitation tasks. Hence we do not need to specify a functional form for our utility function.

#### 5.2.1 Hypotheses: effects of risk aversion

These hypotheses deal with the theoretical predictions of our model. The first hypothesis addresses how behavior responds to exogenous changes in the risk of losing in Round 2.

**Claim 8** *As the prior $p$ increases, subjects switch to protection for higher risks $r$.*

This is a direct test of our model, namely of the monotonicity of the switching curve. The intuition here is that as $p$ increases, the expected payoff from protection decreases relative to exposure. For every $r$, this means that not protecting becomes more attractive and a larger $r$ is needed for the subject to be indifferent.

In order to investigate the effect that risk attitudes have on behavior, we directly measure them using risk elicitation tasks. Our initial challenge is to make inferences about whether the risk aversion measured in our risk elicitation tasks carries over to the main task. As mentioned above, there is evidence in the literature that risk preferences are heavily context dependent. In order to control for this possibility, we include a variety of risk aversion measures in our experimental set-up.

The following hypotheses assume that the risk preferences identified carry over to the main treatment. They follow directly from the propositions in Sect. 3.

**Claim 9** *As risk aversion increases, subjects stop protecting for lower priors p when risk r is high. This would result in a downward shift of the switching curve's upper intercept. In other words, subjects turn fatalistic more easily.*

If having already lost is highly likely, not protecting leads to losing the entire game with very high probability whereas protection retains a small chance of winning. Subjects who dislike risk aim to minimize variation in payoffs. Hence, the small chance of winning is outweighed by saving the certain cost of protection. It therefore requires a lower likelihood of having lost in Round 1 for more risk averse subjects to be indifferent between protecting and not protecting, given that $r$ is high.

Should this hypothesis be rejected, then one of two things is implied. Either risk preferences do not carry over from our risk elicitation tasks, or expected utility theory is not an ideal candidate for explaining preferences in this situation.

**Claim 10** *As risk aversion $\theta$ increases, subjects start protecting for lower risks r when the prior p is low. This would result in a shift to the left of the switching curve's lower intercept.*

Subjects dislike being exposed to risk and hence choose to protect for lower risks of losing Round 2. However, the underlying theory predicts a relatively smaller shift in the lower intercept compared to the upper intercept. Hence, failure to identify an effect may not mean traditional risk aversion measures are a poor predictor of risk aversion in the main task. It could simply be that the effects are too small for our experiment to pick up.

Finally, in Sect. 3 we conjecture that increasing risk aversion may reduce protection overall. Underlying this proposition is that the downward shift of the upper intercept, which decreases protection, outweighs the shift to the left of the lower intercept, which increases protection.

**Claim 11** *As risk aversion $\theta$ increases, the overall number of protection choices decreases.*

This would be an important finding, because it has major implications for health policy. If higher risk aversion increases the probability of an agent choosing not to protect, it might actually have undesirably effects on the spread of an epidemic. On the other hand, rejection of this hypothesis could imply that our model may not be a perfect fit for the underlying preferences.

Regarding all the hypotheses above, failure to identify the effects might not conclusively mean an absence of an effect. Our design necessitates that we observe subjects' choices in a discrete space. The downside to this is that some changes might be too subtle for our data to pick up. Since we cannot differentiate between this and the absence of an effect, we might not be able to firmly reject our hypotheses or reject the model's predictive power.

Having outlined our experimental hypotheses we proceed with detailing the experimental design.

### 5.3 Results

By choosing whether to protect across scenarios in the main task, subjects effectively fill in a 16 × 12 sized grid of choices. We use this grid to trace out subjects' points of switching which allows us to draw a switching curve. To make statements about the relationship between choices and independent observables, we look at several components of subjects' switching curves.

We summarize subjects' displayed switching curves according to three key variables. As dependent variables we use the total amount of times protection was chosen, which makes up the area under the switching curve; the intercept of the switching curve with the "risk of losing in Round 2 being 100%" line, the upper intercept; and the intercept of the switching curve with the "likelihood of having lost Round 1 being 0%" line, the lower intercept. By looking at these key observables, we can determine how changes in independent variables cause shifts in subjects' switching curves. As values for the latter two variables, we measure the actual switching points. For the upper intercept, this is the probability of already having lost in Round 1 at which subjects switch from protection to no protection, given the risk of losing in Round 2 is 100%. Likewise, for the lower intercept, this is the risk of losing in Round 2, for which subjects switch from no protection to protection, given the probability of having lost in Round 1 is 0%

To account for measurement errors, we devise a robustness measure for the intercepts. We measure the average likelihood of already having lost in Round 1, for which subjects switch from protection to no protection, given the risk of losing in Round 2 is 100%, 90% and 80%, for the upper intercept; and the average risk of losing in Round 2, for which subjects switch from no protection to protection, given the likelihood of already having lost is 0%, 5% and 10%, for the lower intercept. We use these averages to test robustness of the results obtained with the actual switching points.

Before we can investigate our theoretical predictions, we need to verify that our model indeed has predictive power. This means we want to find out if subjects make choices according to an implied switching curve, as our model predicts. We carry out regressions in Table 10 of "Appendix G" to test for this. Claim 8 predicts that the likelihood of already having lost in Round 1, that leads subjects to being indifferent between protection and no protection, and the risk of losing in Round 2 have a monotone increasing relationship. Regressing the former on the latter in specification (1) yields a highly significant, positive coefficient, which implies an increasing function. Further, regressing the difference in said likelihoods on risk of becoming infected in specification (2), shows a highly significant, negative coefficient. This implies a decreasing slope and hence concavity. Therefore, in general, subjects display monotonically increasing concave switching curves as our model predicts, verifying Claim 8.

We investigate the theoretical Claims 9–11 by running Ordinary Least Squares (OLS) regressions of our intercepts on the measures of risk aversion and other controls. We find that subjects make choices in accordance with some implied switching curve. Subjects display a continuous threshold above which they switch to protection as the risk of losing in Round 2 increases, just like our model predicts.

Having confirmed above that our model indeed has some predictive power, we investigate the relationship between our main task and the risk aversion elicitation

**Table 1** Baseline regressions by treatment

| | (1) UI | (2) LI | (3) Total protection |
|---|---|---|---|
| IG | 0.203* | − 0.017 | 0.317* |
| | (0.112) | (0.059) | (0.144) |
| Fatalism | 4.651 | 3.735 | 0.418 |
| | (4.116) | (2.160) | (5.294) |
| Male | 3.027 | 0.373 | − 17.846 |
| | (12.427) | (6.523) | (15.984) |
| Masters | − 8.636 | 4.209 | − 21.279 |
| | (11.761) | (6.173) | (15.127) |
| Phd | 3.802 | 6.078 | − 26.051 |
| | (15.121) | (7.937) | (19.449) |
| _cons | 56.959** | 1.330 | 84.474*** |
| | (16.362) | (8.588) | (21.045) |
| $N$ | 34 | 34 | 34 |
| $R^2$ | 0.427 | 0.423 | 0.489 |

Standard errors in parentheses. Each regression also includes session dummies

*$p < 0.05$; **$p < 0.01$; ***$p < 0.001$

measures. Table 1 displays the baseline regressions for our three metrics, the upper intercept (specification (1)), the lower intercept (specification (2)) and the total number of protection choices (specification (3)).

The IG measure has a significant effect on 2 out of these 3 metrics, namely the upper intercept and the total number of protection choices. Furthermore, these effects are of the direction that theory predicts. The IG measure is a significant predictor at the 5% level of the upper intercept, specification (2). Claim 9 predicts that as risk aversion increases, subjects stop protecting for lower likelihoods of having lost in Round 1, given losing in Round 2 is very likely. Our regression (2) confirms that more risk averse subjects turn fatalistic more easily. In specification (3) we identify a positive coefficient for the IG measure on the total number of protection choices. Claim 11 predicts that risk aversion increases protection in the task overall and our result verifies this. These results imply that the IG risk elicitation method is well suited to predict risk attitudes in our main task.

We are unable to find an effect for the lower intercept. The hypothesis in Claim 10 states that increased risk aversion shifts the lower intercept of the switching curve to the left. As mentioned in Sect. 5.2, the lack of any significant effects may be due to a lack of measurement detail in the main task since predicted effects are rather small.

We can infer from this that the IG measure satisfies Claims 8, 9 and 11. Most importantly, there is evidence for the counter-intuitive downward shift in the upper intercept as IG risk aversion increases.

We carry out a series of robustness checks for our results. In Table 2 we display regressions for the upper intercept and lower intercept using the average measures we explained at the beginning of the Results section.

**Table 2** Robustness regressions using average measures

| | (1) Average UI | (2) Average LI |
|---|---|---|
| IG | 0.243* | − 0.034 |
| | (0.102) | (0.074) |
| Fatalism | 5.251 | − 0.889 |
| | (3.735) | (2.698) |
| Male | − 4.474 | − 4.330 |
| | (11.279) | (8.145) |
| Masters | − 14.907 | 2.118 |
| | (10.674) | (7.709) |
| Phd | − 7.359 | 3.583 |
| | (13.724) | (9.911) |
| _cons | 54.336** | 10.010 |
| | (14.850) | (10.725) |
| $N$ | 34 | 34 |
| $R^2$ | 0.491 | 0.375 |

Standard errors in parentheses. Each regression also includes session dummies. The coefficient on IG is significant at the 10% level in specification (5)

$^{*}p < 0.05$; $^{**}p < 0.01$; $^{***}p < 0.001$

#### 5.3.1 Discussion of experimental results

In our experiment we found evidence that supports most of our theoretical results. Increased risk aversion, as measured by the IG, increases implied risk aversion in the main task and increases the total number of protection choices. Most importantly, it shifts the upper intercept downwards, indicating that increased risk aversion does in fact increase the propensity to turn fatalistic. We do not find definitive evidence supporting the theoretical shift in the switching curve's lower intercept, but the problem could lie in the detail with which our main task measures responses. As we briefly discussed, even if we were to change risk aversion from the bottom of the distribution estimated in Holt and Laury (2002) to the top, using the payoffs in the experiment, we would expect to see a change from ca. 15% to 2% risk of becoming infected for the lower intercept. Any change within the distribution will be even smaller meaning our measure over that range (0%, 1%, 5%, 10%, 15%, 20%) might not be able to pick up responses. Therefore noise in the data could lead to no effects being detected, or even false significant effects.

## 6 Conclusion

In this paper, we have demonstrated that individuals' risk attitudes may have non-trivial effects on their propensity to engage in unprotected interactions. In particular, we find that individuals who face sufficiently high risks of infection will become more inclined towards unprotected behavior as they become more risk averse.

Based on the analysis in this paper, we question the common assertion that unprotected behavior is necessarily evidence of risk-seeking preferences. As we have shown, an individual who believes themself to be infected with high probability may find it privately optimal to engage in unprotected exposure. Turning to risk attitudes, we have shown that in a high-prevalence environment, the willingness to engage in unprotected behavior is not necessarily an indication that the individual is risk tolerant, as claimed by Ku et al. (2013) and Lammers and van Wijnbergen (2008). In fact, we find the exact opposite to be the case. Specifically, we show that it may be the least risk tolerant individuals who are the most likely to engage in unprotected behavior. The relationship between risk tolerance and the decision to protect against infection depends on the perceived infection risks, with risk averse individuals being more prone to unprotected exposure in perceived high risk environments.

It is worth emphasizing that although our results may seem very counter-intuitive at first, we have shown that they are in fact based on perfectly standard preferences and are not the result of any extreme or unusual assumptions.

This paper is a first step towards a comprehensive analysis of the effects of risk attitudes on decision making under uncertainty in contexts of asymptomatic infection. It makes clear that risk attitudes may have non-trivial and counter-intuitive effects on individuals' willingness to expose themselves to infection, effects that may carry over to population-wide equilibrium patterns of decision making.

In our experimental analysis, we find evidence in support of our theory in a laboratory setting. We are able to verify the core proposition of the theory in a neutral context. An Investment Game measure of risk aversion corroborates the theoretical prediction that risk aversion increases the propensity to act in a fatalistic manner.

We believe this work provides a solid experimental design that in its general form could be employed in a field experiment. In order to do so, more adequate measures of risk aversion and possibly altruistic preferences would have to be identified.

As any model must be, ours is a simplification of the preferences that may guide behavior and decision-making involving health and disease risks. In practice, people may factor in others' wellbeing and be altruistic or spiteful. Altruism may extend to specific people only or be generalized to the population at large. Our analysis does not cover such cases explicitly and this appears to us to be a worthwhile subject of further study. Toxvaerd (2021) considers altruism in bilateral settings, but no doubt further work in this direction is warranted.

Last, our analysis has been confined to the relationship between risk attitudes and one specific health intervention, namely social distancing (or risk mitigation). Clearly, risk attitudes may also play a role in people's decisions on whether to use other tools of disease mitigation, such as vaccines, antivirals and tests. Having said that, tests and pharmaceutical interventions were unavailable in the early stages of the epidemic and not universally available even now, well into the epidemic. Understanding the effects of risk aversion on basic self-protection, short of these other tools of individual and public health, therefore seems to be of particular importance.

**Data availability** Data available from the corresponding author.

**Open Access** This article is licensed under a Creative Commons Attribution 4.0 International License, which permits use, sharing, adaptation, distribution and reproduction in any medium or format, as long as you give appropriate credit to the original author(s) and the source, provide a link to the Creative Commons licence, and indicate if changes were made. The images or other third party material in this article are included in the article's Creative Commons licence, unless indicated otherwise in a credit line to the material. If material is not included in the article's Creative Commons licence and your intended use is not permitted by statutory regulation or exceeds the permitted use, you will need to obtain permission directly from the copyright holder. To view a copy of this licence, visit http://creativecommons.org/licenses/by/4.0/.

## A. Figures

The figures in this paper are drawn for the CRRA function $u(x) = x^{1-\theta}/(1-\theta)$, with numerical values $x_H^R = 6$, $x_I^R = 1$, $x_H^S = 5.5$ and $x_I^S = 0.5$. Figure 2 shows the cases $\theta_1 = 0$, $\theta_2 = 0.9$ and $\theta_3 = 2$ and Fig. 3 shows the cases $\theta = 0$, $\theta_2 = 0.4$ and $\theta_3 = 0.6$. The left-hand panel of Fig. 3 shows the case $\phi = 0.3$ while the right-hand panel shows the case $\phi = 0.715$.

## B. Proof of Proposition 1

The proofs that follow employ a variation of Theorem 1 in Pratt (1964). For simplicity, we shall make use of the shorthand notation $u(x, \theta)$. Assume that an individual's preferences can be expressed by a utility function $u(x)$ where $x$ is the payoff. Let $\theta(x)$ be the coefficient of local risk aversion. An individual is indifferent between exposure and protection if and only if $U^R = U^S$. This will occur at $r^*$.

First, let

$$U^R(\theta_1) \equiv \left(1 - p^R\right) u\left(x_H^R, \theta_1\right) + p^R u\left(x_I^R, \theta_1\right) \tag{10}$$

Since $p^R = p + (1-p)r$, it follows that

$$\lim_{p\to 0} U^R(\theta_1) = (1-r)\, u\left(x_H^R, \theta_1\right) + r u\left(x_I^R, \theta_1\right) \tag{11}$$

Similarly, let

$$U^S(\theta_1) \equiv \left(1 - p^S\right) u\left(x_H^S, \theta_1\right) + p^S u\left(x_I^S, \theta_1\right)$$

Then it follows that

$$\lim_{p\to 0} U^S(\theta_1) = u\left(x_H^S, \theta_1\right) \tag{12}$$

For switching, it is hence required that

$$\lim_{p\to 0} U^R(\theta_1) = \left(1 - r_1^*\right) u\left(x_H^R, \theta_1\right) + r_1^* u\left(x_I^R, \theta_1\right) = u\left(x_H^S, \theta_1\right) = \lim_{p\to 0} U^S(\theta_1) \tag{13}$$

By the definition of risk aversion, $x_H^S$ must be the certainty equivalent of the lottery $\left(x_H^R, x_I^R; 1-r_1^*, r_1^*\right)$. Denote this certainty equivalent by $CE_1 \equiv x_H^S$ and note that

$$u\left(CE_1, \theta_1\right) = u\left(x_H^S, \theta_1\right) = \left(1-r_1^*\right) u\left(x_H^R, \theta_1\right) + r_1^* u\left(x_I^R, \theta_1\right) \tag{14}$$

Now consider an increase in the coefficient of risk aversion to $\theta_2(x) > \theta_1(x)\, \forall x$. Keeping $r = r_1^*$ fixed, we now have that

$$\lim_{p\to 0} U^R(\theta_2) = \left(1-r_1^*\right) u\left(x_H^R, \theta_2\right) + r_1^* u\left(x_I^R, \theta_2\right) \tag{15}$$

From risk aversion, it follows that the certainty equivalent of the gamble $\left(x_H^R, x_I^R;\right.$ $\left.1-r_1^*, r_1^*\right)$ must be lower under coefficient $\theta_2(x)$ than under $\theta_1(x)$. Thus

$$CE_2 < CE_1 = x_H^S \tag{16}$$

and it follows that

$$u\left(x_H^S, \theta_2\right) > u\left(CE_2, \theta_2\right) = \left(1-r_1^*\right) u\left(x_H^R, \theta_2\right) + r_1^* u\left(x_I^R, \theta_2\right) \tag{17}$$

Again, switching requires that

$$u\left(x_H^S, \theta_2\right) = \left(1-r_2^*\right) u\left(x_H^R, \theta_2\right) + r_2^* u\left(x_I^R, \theta_2\right) \tag{18}$$

Since $x_H^R > x_H^S > x_I^R$, it follows directly that

$$r_2^* < r_1^* \tag{19}$$

This completes the proof □

## C. Proof of Proposition 3

It is straightforward to verify that

$$\lim_{r\to 1} U^R(\theta_1) = u\left(x_I^R, \theta_1\right) \tag{20}$$

For switching it is hence required that,

$$\lim_{r\to 1} U^R(\theta_1) = u\left(x_I^R, \theta_1\right) = \left(1-p_1^*\right) u\left(x_H^S, \theta_1\right) + p_1^* u\left(x_I^S, \theta_1\right) = \lim_{r\to 1} U^S(\theta_1) \tag{21}$$

By risk aversion, $x_I^R$ is the certainty equivalent of the lottery $\left(x_H^S, x_I^S; 1-p_1^*, p_1^*\right)$. Denote this certainty equivalent by $CE_1 = x_I^R$ and note that

$$u\left(CE_1, \theta_1\right) = u\left(x_I^R, \theta_1\right) = \left(1-p_1^*\right) u\left(x_H^S, \theta_1\right) + p_1^* u\left(x_I^S, \theta_1\right) \tag{22}$$

Now consider an increase in the coefficient of risk aversion to $\theta_2(x) > \theta_1(x)\,\forall x$. Now, we have that

$$\lim_{r\to 1} U^S(\theta_2) = \left(1-p_1^*\right) u\left(x_H^S, \theta_2\right) + p_1^* u\left(x_I^S, \theta_2\right) \tag{23}$$

By risk aversion, it must be that the certainty equivalent of the gamble $\left(x_H^S, x_I^S; 1-p_1^*, p_1^*\right)$ must be lower under coefficient $\theta_2(x)$ than under $\theta_1(x)$. Thus

$$CE_2 < CE_1 = x_I^R \tag{24}$$

and it follows that

$$u\left(x_I^R, \theta_2\right) > u_i\left(CE_2, \theta_2\right) = \left(1-p_1^*\right) u\left(x_H^S, \theta_2\right) + p_1^* u\left(x_I^S, \theta_2\right) \tag{25}$$

Indifference requires that

$$u\left(x_I^R, \theta_2\right) = \left(1-p_2^*\right) u\left(x_H^S, \theta_2\right) + p_2^* u\left(x_I^S, \theta_2\right) \tag{26}$$

Since $x_H^S > x_I^R > x_I^S$, it follows directly that

$$p_2^* < p_1^* \tag{27}$$

This completes the proof □

## D. Proof of Proposition 5

Consider first the expression for the expected utility from an unprotected encounter, namely

$$U^R = \left(1-p^R\right) u\left(x_H^R, \theta_1\right) + p^R u\left(x_I^R, \theta_1\right) \tag{28}$$

Recall that $p^R = p + (1-p)r$ and therefore

$$\lim_{r\to 1} U^R = u\left(x_I^R, \theta_1\right) \tag{29}$$

Similarly,

$$\lim_{r\to 1} U^S = (1-p)\left[(1-\phi)\,u\left(x_H^S,\theta_1\right)+\phi u\left(x_I^S,\theta_1\right)\right]+pu\left(x_I^S,\theta_1\right) \tag{30}$$

For switching it is hence required that under imperfect protection,

$$\begin{aligned} U^S &= \left(1-p_1^*\right)\left[(1-\phi)\,u\left(x_H^S,\theta_1\right)+\phi u\left(x_I^S,\theta_1\right)\right]+p_1^* u\left(x_I^S,\theta_1\right)\\ &= u\left(x_I^R,\theta_1\right)=U^R \end{aligned} \tag{31}$$

We follow a similar approach as in the proof of Proposition 3. Let $\theta_2(x) > \theta_1(x)\,\forall x$. By continuity of $u(x)$ there exists a payoff $x'$, such that $x_H^S > x' > x_I^R > x_I^S$ and

$$(1-\phi)\,u\left(x_H^S,\theta_1\right)+\phi u\left(x_I^S,\theta_1\right)=u\left(x',\theta_1\right) \tag{32}$$

Substituting this into (31) we obtain

$$\left(1-p_1^*\right)u\left(x',\theta_1\right)+p_1^* u\left(x_I^S,\theta_1\right)=u\left(x_I^R,\theta_1\right) \tag{33}$$

From concavity of $u$, it follows that

$$\left(1-p_1^*\right)u\left(x',\theta_2\right)+p_1^* u\left(x_I^S,\theta_2\right)<u\left(x_I^R,\theta_2\right) \tag{34}$$

Similarly, we have

$$(1-\phi)\,u\left(x_H^S,\theta_2\right)+\phi u\left(x_I^S,\theta_2\right)<u\left(x',\theta_2\right) \tag{35}$$

It follows that there exists a $\phi' < \phi$ for which

$$\left(1-\phi'\right)u\left(x_H^S,\theta_2\right)+\phi' u\left(x_I^S,\theta_2\right)=u\left(x',\theta_2\right) \tag{36}$$

Substituting for $u\left(x',\theta_2\right)$ in (34), we obtain

$$\frac{u\left(x_H^S,\theta_2\right)-u\left(x_I^R,\theta_2\right)}{u\left(x_H^S,\theta_2\right)-u\left(x_I^S,\theta_2\right)}<p_1^*+\phi'-p_1^*\phi' \tag{37}$$

Since $\phi' < \phi$ we know that

$$\frac{u\left(x_H^S,\theta_2\right)-u\left(x_I^R,\theta_2\right)}{u\left(x_H^S,\theta_2\right)-u\left(x_I^S,\theta_2\right)}<p_1^*+\phi-p_1^*\phi \tag{38}$$

Indifference requires that $U^R = U^S$ and hence

$$\frac{u\left(x_H^S, \theta_2\right) - u\left(x_I^R, \theta_2\right)}{u\left(x_H^S, \theta_2\right) - u\left(x_I^S, \theta_2\right)} = p_2^* + \phi - p_2^* \phi \tag{39}$$

It follows directly that

$$p_2^* < p_1^* \tag{40}$$

This completes the proof □

## E. Proof of Proposition 6

We will show that

$$\lim_{p \to 0} \frac{dr^*(p, \phi)}{d\theta} \leq 0 \; \forall \phi \leq \bar{\phi}, \quad \lim_{p \to 0} \frac{dr^*(p, \phi)}{d\theta} > 0 \; \forall \phi > \bar{\phi} \tag{41}$$

We have that

$$\lim_{p \to 0} U^R(\theta_1) = (1 - r)\, u\left(x_H^R, \theta_1\right) + ru\left(x_I^R, \theta_1\right) \tag{42}$$

Similarly,

$$\lim_{p \to 0} U^S(\theta_1) = (1 - \phi r)\, u\left(x_H^S, \theta_1\right) + \phi ru\left(x_I^S, \theta_1\right) \tag{43}$$

Under imperfect protection, switching amounts to

$$\begin{aligned} U^S(\theta_1) &= \left(1 - \phi r_1^*\right) u\left(x_H^S, \theta_1\right) + \phi r_1^* u\left(x_I^S, \theta_1\right) \\ &= \left(1 - r_1^*\right) u\left(x_H^R, \theta_1\right) + r_1^* u\left(x_I^R, \theta_1\right) = U^R(\theta_1) \end{aligned} \tag{44}$$

For completeness, the threshold failure probability is given by

$$\bar{\phi} = \frac{\left(u\left(x_H^S, \theta_2\right) - u\left(x_I^S, \theta_2\right)\right) - \frac{u\left(x_H^S, \theta_1\right) - u\left(x_I^S, \theta_1\right)}{u\left(x_H^R, \theta_1\right) - u\left(x_H^S, \theta_1\right)}\left(u\left(x_H^R, \theta_2\right) - u\left(x_H^S, \theta_2\right)\right)}{\left(u\left(x_H^R, \theta_2\right) - u\left(x_I^R, \theta_2\right)\right) - \frac{u\left(x_H^R, \theta_1\right) - u\left(x_I^R, \theta_1\right)}{u\left(x_H^R, \theta_1\right) - u\left(x_H^S, \theta_1\right)}\left(u\left(x_H^R, \theta_2\right) - u\left(x_H^S, \theta_2\right)\right)} \tag{45}$$

We follow a similar approach as in the previous proofs. Let $\theta_2(x) > \theta_1(x) \, \forall x$. By continuity of $u(x)$, there exists a payoff $x'$, such that $x_H^S > x'' > x_I^R > x_I^S$ and

$$\left(1 - \phi r_1^*\right) u\left(x_H^S, \theta_1\right) + \phi r_1^* u\left(x_I^S, \theta_1\right) = u\left(x'', \theta_1\right) \tag{46}$$

Equation (44) then implies that

$$\left(1-r_1^*\right)u\left(x_H^R,\theta_1\right)+r_1^*u\left(x_I^R,\theta_1\right)=u\left(x'',\theta_1\right) \tag{47}$$

We know by risk aversion that

$$\left(1-r_1^*\right)u\left(x_H^R,\theta_2\right)+r_1^*u\left(x_I^R,\theta_2\right)<u\left(x'',\theta_2\right) \tag{48}$$

and also that

$$\left(1-\phi r_1^*\right)u\left(x_H^S,\theta_2\right)+\phi r_1^*u\left(x_I^S,\theta_2\right)<u\left(x'',\theta_2\right) \tag{49}$$

But (49) implies that there exists a $\phi'' < \phi$ such that

$$\left(1-\phi'' r_1^*\right)u\left(x_H^S,\theta_2\right)+\phi'' r_1^*u\left(x_I^S,\theta_2\right)=u\left(x'',\theta_2\right) \tag{50}$$

Substituting for $u\left(x',\theta_2\right)$ into (48), we obtain the following inequality:

$$\frac{u\left(x_H^R,\theta_2\right)-u\left(x_H^S,\theta_2\right)}{u\left(x_H^S,\theta_2\right)-u\left(x_I^S,\theta_2\right)}<\left[\frac{u\left(x_H^R,\theta_2\right)-u\left(x_I^R,\theta_2\right)}{u\left(x_H^S,\theta_2\right)-u\left(x_I^S,\theta_2\right)}-\phi''\right]r_1^* \tag{51}$$

Recall that $\phi'' < \phi$. If we let $\phi'' \to \phi$, the right-hand side of (51) decreases. Depending on the relative magnitudes of $\phi''$ and $\phi$ , one of two possibilities arises. Either we have

$$\frac{u\left(x_H^R,\theta_2\right)-u\left(x_H^S,\theta_2\right)}{u\left(x_H^S,\theta_2\right)-u\left(x_I^S,\theta_2\right)}\le\left[\frac{u\left(x_H^R,\theta_2\right)-u\left(x_I^R,\theta_2\right)}{u\left(x_H^S,\theta_2\right)-u\left(x_I^S,\theta_2\right)}-\phi\right]r_1^* \tag{52}$$

or we have

$$\frac{u\left(x_H^R,\theta_2\right)-u\left(x_H^S,\theta_2\right)}{u\left(x_H^S,\theta_2\right)-u\left(x_I^S,\theta_2\right)}>\left[\frac{u\left(x_H^R,\theta_2\right)-u\left(x_I^R,\theta_2\right)}{u\left(x_H^S,\theta_2\right)-u\left(x_I^S,\theta_2\right)}-\phi\right]r_1^* \tag{53}$$

That is, if $\phi$ is only slightly larger than $\phi''$, then the original inequality remains valid. However, if $\phi$ is significantly larger than $\phi''$, then the inequality is reversed.

Indifference requires the inequalities (52) and (53) to hold with equality at $r_2^*(0,\phi)$. Hence changing $r_1^*$ to $r_2^*$ in the inequalities will lead to strict equality. Since the right-hand side is strictly increasing in $r$, it follows that

$$r_2^* \le r_1^* \tag{54}$$

for (52) and

$$r_2^* > r_1^* \tag{55}$$

for (53). This completes the proof □

## F. Experimental analysis

### Additional details on experimental design

#### Controlling for understanding

Our main task is rather complex by experimental economics standards. When pilot testing the experiment, we realized that some subjects struggled with the computation of payoffs. Therefore relying solely on the instructions is risky. In order to ensure that subjects fully understand the main task, two further control measures were implemented.

The main task is preceded by interactive examples for subjects to practice with. Here subjects can make their choices and an example walks them through the implications of their choice. Furthermore, after said examples, subjects are asked to complete a quiz. This quiz comes in the form of another example where subjects have to fill in blanks. The quiz contains 10 questions that ask subjects to correctly identify probabilities and compute payoffs for the scenario. Failing this quiz will result in another quiz once it has been indicated to subjects which answers they got wrong previously. Failing the second quiz sends a notification to the experimenter who will then personally walk the subject through a third and final quiz. Subjects' performance in the quizzes is recorded so they may be flagged for possibly not having understood the task. We control for understanding in some robustness checks.

#### Computation of odds

The overall odds of losing the game are not necessarily straightforward to compute given combinations of $r$ and $p$. Throughout the sessions, subjects are unable to communicate and do not have access to mobile phones, calculators or pen and paper to make calculations. In order to control for subjects' choices being based on actual odds of infection rather than influenced by different levels of mathematical ability, odds are computed for the subject. They are displayed at the bottom of the screen when the mouse cursor is moved over the scenario in question. In order to control for whether this display of odds has any effect on behavior, subjects also filled out a subset of scenarios (6 times 12) where no such aid was given. Using a paired t-test, we find there is a difference at the 10% significance level between the total number of "safe" choices when odds are displayed to when they are not ($t\,(33) = 1.523$, $P = 0.068$). When repeating the test by whether subjects have a science or mathematical degree background ($t\,(31) = -0.33$, $P = 0.747$) or an arts and humanities background ($t\,(26) = 1.29$, $P = 0.104$), the difference is only borderline significant for the latter. Assuming the first group to be more mathematically inclined, this lets us conclude that the difference in the responses is due to subjects finding it difficult to calculate the odds themselves, giving support to our design choice. The difference between the total

**Table 3** Summary statistics—control measures

| Variable | Mean | (SD) | Min. | Max. | N |
|---|---|---|---|---|---|
| Total number of protection choices | 94.49 | (35.22) | 12 | 159 | 70 |
| Risky choices in MPL (out of 10) | 3.88 | (1.52) | 1 | 7 | 67 |
| % subjects MPL consistent with EUT | 0.96 | (0.2) | 0 | 1 | 70 |
| Blocks collected in BRET (out of 100) | 42.73 | (13.72) | 10 | 75 | 70 |
| Pence invested in IG (out of 200) | 109.63 | (61.16) | 0 | 200 | 70 |
| Fatalism measure (out of 4) | 1.94 | (0.93) | 1 | 4 | 70 |

**Table 4** Means by gender

| Variable | Female | Male | *p* value (t-test) |
|---|---|---|---|
| Pence invested in IG (of 200) | 92.65 (44.71) | 136.67 (73.90) | 0.002 |
| Risky choices in MPL (of 10) | 3.76 (1.53) | 4.08 (1.52) | 0.405 |
| Blocks collected in BRET | 41.91 (11.64) | 44.04 (16.67) | 0.531 |
| Fatalism measure (of 4) | 1.02 (0.94) | 0.81 (0.92) | 0.366 |
| Protection offered in AET (of 10) | 7.37 (3.46) | 7.26 (3.63) | 0.897 |
| *N* | 43/41 | 27/26 | |

number of "safe" choices when odds are displayed to when they are not, disappears in a disease context setting ($t$ (59) $= 0.81$, $P = 0.419$), more on this below.

## Risk aversion and fatalism

First, note that all risk attitude measures in this paper are expressed in terms of risky behavior. That means that the larger the values of the IG, MPL and BRET, the more risk taking is displayed by the subject.

Table 3 summarizes our risk attitude and fatalism measures. 96% of subjects (all but 3) make choices in the MPL task that are consistent with expected utility theory.[15] This is similar to what is observed in Holt and Laury (2002) and Lonnqvist et al. (2014). The average number of risky choices in the MPL task (3.9) is close to that observed in Crosetto and Filippin (2013a, b). It is slightly below the 4.8 average recorded in Holt and Laury (2002), however, we can attribute this to larger compensation in our experiment which has been shown to decrease risk taking in this task. The average number of blocks collected in the BRET lies within the range of averages observed in Crosetto and Filippin (2013a, b). Finally, the average investment in the IG is 54.8% of the endowment. This is below investment levels of 63.6% observed in Crosetto and Filippin (2013a, b). Again, this can be attributed to larger compensation. The 1.94 mean response to whether subjects think they "have little control over their life" on a scale of 1–4 is close to the 1.81 recorded in Shapiro and Wu (2011).

15 In regressions using the MPL we omit inconsistent observations.

Table 4 shows means across gender. In general males take on more risk than females. We observe no significant gender effects for the MPL or BRET measure. However, there appears to be a significant gender effect for the IG measure. These findings are consistent with findings in Holt and Laury (2002), Charness and Gneezy (2010) and Crosetto and Filippin (2013a, b).

Looking at correlations between our measures, the MPL measure is strongly correlated with the IG measure at a 0.1% significance. The MPL is also significantly correlated with the BRET measure at 5% significance. There appears to be only weak correlation between the IG and BRET measure. Our measure for fatalism is exclusively correlated with the BRET measure at a 5% significance level. These findings are slightly at odds with the literature. Crosetto and Filippin (2013a, b) indirectly show low correlation across the MPL, IG and BRET measures. Bruner (2009) and Reynaud and Couture (2012) show low correlation across different measures in general, although they do not offer a direct comparison of the measures presented here.

At this point a word of caution is in order. To avoid sample selection biases, we compare the means of the above variables across the two treatments (the main neutral game show treatment and an alternative disease-context treatment, described in detail below). As can be seen in Table 9, we record a significant difference in the distribution of the number of risky choices in the MPL across treatments for both a two-tailed t-test ($t\,(65) = 2.66$, $P = 0.005$) and a Mann–Whitney test ($z = 2.28$, $P = 0.023$). Neither the IG nor the BRET display such biases. It appears that subjects in the game show treatment made significantly riskier choices in the MPL. The MPL and our main task, especially the game show treatment are similar in certain respects, especially in the way the prospects of winning and losing are displayed. See Figs. 6 and 7. Since the risk aversion measure is elicited after the main task is completed, we are concerned that the treatment might affect choices in the MPL. Indeed, we find that the difference in the sample is significant when we look at subjects that played the MPL in direct succession to the main task ($t\,(25) = 2.42$, $P = 0.011$) but not significant if there was another task, either the IG or the BRET, in between the two ($t\,(38) = 2.42$, $P = 0.174$).

Furthermore, we find a significant difference in the MPL measure for people whose university major was in a field with a mathematical or science background compared to those with arts or humanities degrees ($t\,(65) = -1.96$, $P = 0.027$). The MPL has a reputation in the literature for being rather complex, which could cause this difference, meaning the MPL could be contaminated by differences in mathematical ability. Because of these concerns, we decided to exclude the MPL from our regressions.

We also need to be aware that the BRET is a fairly new measure and its properties are yet to be examined more thoroughly by the literature. Its correlation with the more established measures is limited but it is uniquely correlated with our fatalism measure. Furthermore, it predicts statistically significant larger amounts of risk loving then either of the other two measures. Using the BRET, 14 subjects (20%) are classified as risk lovers. An additional 14% are classified as risk neutral. This compares to 22% of subjects displaying choices that indicated either risk neutrality or risk loving combined in the IG ($t\,(70) = 1.47$, $P = 0.073$) and 19% of subjects acting risk neutral and only one subject acting risk loving in the MPL ($t\,(67) = 2.19$, $P = 0.016$). There is a significant decrease in risk aversion, as estimated using a CRRA specification, between the MPL and the BRET ($t$ (67) $= 6.15$, $P = 0.000$) and the IG and the BRET ( $t$ (70) $= 1.74$, $P = 0.042$). There is a chance the BRET picks up some other factors than, or additional to, pure risk aversion. While our experimental results are robust when the BRET is included (see Table 5), the BRET itself is not significant in any regression and its effect changes direction across treatments. As outlined in Sect. 5.1, the BRET is a not very well explored method of risk elicitation. Without further investigation we decide to use the BRET as a robustness measure only and stick to the more established measures in our main analysis.

### Investigating framing effects

When first designing the experiment, we were concerned about framing effects from presenting the tasks in a disease context. Individuals may treat health related risks very differently from monetary risks meaning that traditional risk elicitation measures may not accurately measure risk attitudes. Further, the context of an epidemic may alter behavior in a way that is not captured by the theory. Dealing with infectious diseases might invoke a feeling of shame, or disgust in the subject that can lead to them losing faith or trust in self-worth. In order to test our theory free from these biases, we must present the task in a neutral context that avoids priming. We therefore opted for the neutral/monetary context setting of the game show. One can consider this the first stage of testing the model's predictions. If they do not hold in a neutral context, then their applicability to a context such as health is in question. Upon passing this initial stage, one can then move on to ascertain whether the model has explanatory power in a more applied context. Therefore, to investigate framing effects, we also provided another treatment where instructions are framed in an explicit disease setting with an unspecified asymptomatic disease.

### Main task (disease context)

Subjects are told that they are at risk of infection with a potentially fatal but non-specified disease. They are also told that the disease is asymptomatic and hence they do not know whether they are already infected. Being healthy is valued at £6. So a healthy individual would earn £6. Should the subject become, or is in fact already, infected they would lose £5.5. So an infected individual would earn £0.5. Furthermore, subjects can spend £0.5 on protection that will eliminate all risk of becoming infected. This protection is, however, ineffective against already being infected. The way the task is conducted and presented to subjects remains the same.

As we show below, changing the context to mimic disease outbreaks can have a significant effect on risk attitudes and underlying preferences.

### Treatment hypotheses: framing and context effects

We set up the following hypotheses about how we expect context to affect behavior.

**Table 5** Robustness regressions using BRET

| | Game show treatment | | | | Disease treatment | | | |
|---|---|---|---|---|---|---|---|---|
| | (1) Est. risk aversion | (2) UI | (3) LI | (4) Total protection | (5) Est. risk aversion | (6) UI | (7) LI | (8) Total protection |
| IG | − 0.003* | 0.223* | 0.011 | 0.341* | − 0.001 | 0.022 | 0.010 | − 0.086 |
| | (0.001) | (0.120) | (0.060) | (0.154) | (0.001) | (0.089) | (0.026) | (0.114) |
| BRET | 0.000 | 0.259 | 0.370 | 0.311 | 0.006 | − 0.703 | − 0.262 | − 0.370 |
| | (0.004) | (0.465) | (0.233) | (0.599) | (0.004) | (0.455) | (0.131) | (0.582) |
| Fatalism | − 0.078* | 3.886 | 2.643 | − 0.500 | − 0.054 | 5.446 | 3.426 | − 1.602 |
| | (0.035) | (4.399) | (2.202) | (5.663) | (0.074) | (9.272) | (2.663) | (11.862) |
| Male | 0.064 | − 1.018 | − 5.398 | − 22.695 | − 0.059 | 1.684 | − 2.902 | 5.535 |
| | (0.116) | (14.558) | (7.289) | (18.743) | (0.091) | (11.425) | (3.281) | (14.617) |
| Masters | 0.087 | − 8.349 | 4.618 | − 20.936 | 0.051 | − 8.577 | 4.341 | − 6.174 |
| | (0.095) | (11.952) | (5.984) | (15.388) | (0.111) | (13.850) | (3.978) | (17.720) |
| PhD | 0.031 | 6.650 | 10.139 | − 22.638 | 0.054 | − 22.810 | − 1.047 | − 2.160 |
| | (0.129) | (16.181) | (8.102) | (20.833) | (0.141) | (17.712) | (5.087) | (22.660) |
| _cons | 0.660** | 45.352 | − 15.225 | 70.564 | 0.265 | 106.025*** | 15.513* | 133.568*** |
| | (0.213) | (26.647) | (13.341) | (34.306) | (0.192) | (24.124) | (6.928) | (30.863) |
| $N$ | 34 | 34 | 34 | 34 | 36 | 36 | 36 | 36 |
| $R^2$ | 0.571 | 0.435 | 0.483 | 0.495 | 0.116 | 0.172 | 0.247 | 0.114 |

Standard errors in parentheses. Each regression also includes session dummies. The coefficient on BRET is significant at the 10% level in specification (7)
$*p < 0.05$; $**p < 0.01$; $***p < 0.001$

**Claim 12** *Subjects behave identically in both the game show treatment and the disease treatment. We expect to see no significant difference in the average number of protection choices across treatments.*

Failure to reject this claim would suggest that our model is indeed applicable to both settings and subjects behave, on average, in the same way across contexts. Should we be able to reject the hypothesis, one possible explanation might be that subjects have an inherent bias towards protection in the disease treatment. In this case, we would observe a significantly larger average number of protection choices in this treatment. This bias could be driven by a variety of underlying factors. The simplest explanation is that subjects do not like the idea of being infected with diseases and hence place an additional emotional value on "health" (or an emotional cost on "infection") over and above the monetary incentives provided in the experiment. Therefore the costs and benefits of the disease treatment and the game show treatment may not be perfectly aligned. Such a bias is easily addressed in the model by adjusting the values of $x_h^a$ across infection states. An alternative possible bias is that the action of protection might hold some intrinsic value. This could be, for instance, related to the subject feeling more in control. In order to address such a potential bias and inherent fatalistic tendencies, we control for subjects' perceived level of control about their life. We use a measure proposed by Shapiro and Wu (2011).

Our first hypothesis can be analysed in more detail by looking at the implied changes to subjects' switching curves. To be precise, we look at how the shape of the switching curve changes across treatments. The next two hypotheses follow directly from the first one. They are necessary but not sufficient for the first hypothesis to be true.

**Claim 13** *For low priors $p$, switching points (between protection and no protection) are the same in the game show treatment and the disease treatment.*

What this claim implies is that fixing the likelihood of already being infected, subjects, on average, start to protect at the switching point across treatments, indicating no context bias. If it is possible to reject this hypothesis, it could be that subjects display significantly different preferences across contexts. It is possible that subjects start protecting for lower risks of becoming infected in the disease treatment for the above mentioned reasons associated with the context of diseases and health.

**Claim 14** *For high risks $r$, the switching point (between protection and no protection) is the same in the game show treatment and the disease treatment.*

This implies that fixing the infection risk, subjects, on average, protect up to the same likelihood of already being infected across treatments. Apart from providing evidence for a framing effect, rejecting this hypothesis could have significant implications for the importance of rational fatalism. If subjects are biased towards choosing protection in the disease treatment and therefore continue to protect for higher likelihoods of already being infected, they are less likely to respond in a fatalistic fashion. This would reduce the importance of rational fatalism. If people place large emotional value on not being sick and care relatively less about the actual payoffs, then our model needs to account for this. However, if the effect works in the other direction, this could be interpreted as the context giving them the feeling of hopelessness. Actual fatalism could kick in.

So far, we have attributed potential effects to changes in the payoff structure. Alternatively, such a treatment effect could result from a change in the underlying preferences across contexts. For example, risk preferences may differ across treatments. Evidence from the literature suggests that risk preferences can be highly context dependent (Dohmen et al. 2005, 2011; Szrek et al. 2012). A difference in risk preference across contexts could have drastic effects, such as having entirely different utility specifications in different contexts. Or it could imply a small change such as subjects having different coefficients of risk aversion, while still having the same utility function across contexts. One approach could be test for whether the implied risk aversion elicited in our main task differs across treatments. A significant difference in the sample risk aversion estimates would imply a systematic risk attitude change in the context of diseases. This would require us to specify the functional form of the utility function. However, even from this we would not be able to infer from this result alone whether this means that subjects have different utility specifications or simply different levels of risk aversion across contexts. Trying to distinguish between the two would involve estimating the fit of various utility functions in our data, which is beyond the scope of this paper. Furthermore, failure to reject the hypothesis may not imply the absence of an effect, but merely that this effect is non-systematic. If subjects adjust their risk preferences across contexts in a way that means more risk aversion for some and more risk loving for others, we will not be able to find a treatment effect. We therefore refrain from this analysis here.

## Treatment effects

In this section we show that there is little evidence that context has an effect on the observed protection choices. We can neither establish a treatment effect for the overall level of protection choices made nor the level of the intercepts.

The results of non-parametric tests for all metrics are displayed in Table 6. We find no statistical difference in the overall number of protection choices across treatments ($t\,(68) = -0.59$, $P = 0.56$). Similarly, we use non-parametric tests to look for differences in the level of curves' intercepts. For both the lower intercept ($t\,(68) =$

**Table 6** Summary of non-parametric tests

| Metric | t-test | | Mann–Whitney | |
|---|---|---|---|---|
| | t | *p* value | z | *p* value |
| Total of protection choices | −0.591 | 0.556 | −0.541 | 0.589 |
| Lower intercept | 0.974 | 0.333 | 0.287 | 0.774 |
| Upper intercept | 1.11 | 0.272 | 1.088 | 0.277 |
| Lower intercept (Avr) | 1.83 | 0.072 | 1.422 | 0.155 |
| Upper intercept (Avr) | 1.091 | 0.279 | 0.852 | 0.394 |
| NLS-estimate of $\theta$ | −0.648 | 0.519 | −0.623 | 0.533 |
| *N* | 70 | | 70 | |

0.97, $P = 0.33$) and the upper intercept ($t\,(68) = 1.1$, $P = 0.27$), no significant difference across treatments can be found.

To check for robustness of these results, we repeat the tests using the average measure over the last three choices closest to the respective intercept. Using this robustness measure we find a significant difference in means for the lower intercept across treatments at a 5% significance level ( $t\,(68) = 1.8$, $P = 0.04$). This suggests that under the disease treatment, subjects choose to protect for lower risks, given low priors. But the evidence is not conclusive. We fail to identify a similar effect for the upper intercept ($t\,(68) = 1.1$, $P = 0.28$).

Claim 12 is concerned with the average total number of times subjects choose to protect, whereas Claims 13 and 14 are concerned with the levels of protection for fixed values of the risks and priors, respectively. The claims state that there are no significant differences across the two treatments and hence no framing effects. We fail to reject Claims 12 and 14 .

In light of the inconclusive evidence concerning Claim 13, we investigate the treatment effect further using regression analysis. Specifications (1) to (3) in Table 7 present regressions for all metrics mentioned above controlling for treatment.

We indeed find a significant negative treatment effect on the lower intercept, using both the actual level in specification (3) and a robustness test in specification (7). This backs up findings from the non-parametric test using the average robustness measure. A potential explanation for this effect is that given subjects believe they are likely to be healthy, the context of diseases creates an emotional cost associated with infection which leads to protection for lower risks than in the neutral context. We can confidently reject Claim 13.

In summary, we only find weak evidence that context affects the shape of the switching curves and therefore alters behavior in a significant way. The suggestive evidence we do find is in the predicted direction. The disease context shifts the lower intercept to the left. However, while we do observe a treatment effect in one dimension, we fail to reject three out of four Claims. This does not imply that context has no effect. Context could have effects on choices that are not reflected in the actual number of times protection is chosen, but as we will see in the next subsection, context can affect behavior by changing the underlying risk preferences.

#### Impact on theoretical predictions

We find no significant effects for any risk attitude metrics in the disease treatment. There are at least three interpretations of these result. One, framing affects risk attitudes and underlying preferences across different contexts, which is in line with the literature. The chosen risk elicitation tasks may not make for good predictors of risk attitudes in the disease context and one might need to measure risk aversion differently. A second interpretation is that our theory does not apply to the infectious disease context. Possible explanations could be that altruism emerges as a main driver of behavior in the context of diseases, which could be tested for by using a specifically designed altruism elicitation task for the domain of losses. See Toxvaerd (2021) for recent work on altruistic preferences in disease contexts. Finally, it could be that a hypothetical lab experiment does not sufficiently capture real world behavior choices.

**Table 7** Pooled and robustness regressions using BRET

| | Pooled data | | | | | | | |
|---|---|---|---|---|---|---|---|---|
| | (1) Est. risk aversion | (2) UI | (3) LI | (4) Total protection | (5) Est. risk aversion | (6) UI | (7) LI | (8) Total protection |
| IG | −0.001* | 0.071 | −0.001 | 0.045 | −0.001* | 0.075 | −0.001 | 0.048 |
| | (0.000) | (0.060) | (0.022) | (0.077) | (0.000) | (0.060) | (0.022) | (0.078) |
| BRET | | | | | 0.005* | −0.419 | −0.047 | −0.349 |
| | | | | | (0.002) | (0.307) | (0.115) | (0.397) |
| Fatalism | −0.053 | 2.707 | 2.929 | −1.101 | −0.076* | 4.712 | 3.155 | 0.569 |
| | (0.034) | (4.191) | (1.546) | (5.377) | (0.035) | (4.409) | (1.651) | (5.713) |
| Male | −0.056 | 5.597 | −2.252 | 5.349 | −0.079 | 7.549 | −2.032 | 6.974 |
| | (0.062) | (7.733) | (2.852) | (9.922) | (0.061) | (7.804) | (2.923) | (10.113) |
| Masters | 0.109 | −11.736 | 5.406 | −22.383* | 0.113 | −12.088 | 5.366 | −22.676* |
| | (0.068) | (8.500) | (3.135) | (10.906) | (0.066) | (8.437) | (3.160) | (10.933) |
| Phd | 0.066 | −14.027 | 3.014 | −18.317 | 0.108 | −17.531 | 2.618 | −21.235 |
| | (0.089) | (11.055) | (4.077) | (14.184) | (0.089) | (11.263) | (4.218) | (14.596) |
| Disease treatment | −0.118 | 9.267 | −14.438* | 30.367 | −0.151 | 11.995 | −14.130* | 32.638 |
| | (0.134) | (16.596) | (6.120) | (21.294) | (0.131) | (16.586) | (6.212) | (21.494) |
| _cons | 0.708*** | 58.139** | 18.229** | 74.020** | 0.522** | 73.884*** | 20.006* | 87.130** |
| | (0.138) | (17.139) | (6.321) | (21.991) | (0.162) | (20.533) | (7.690) | (26.608) |
| $N$ | 70 | 70 | 70 | 70 | 70 | 70 | 70 | 70 |
| $R^2$ | 0.229 | 0.186 | 0.318 | 0.181 | 0.285 | 0.214 | 0.320 | 0.193 |

Standard errors in parentheses. Each regression also includes session dummies. The coefficient on Fatalism is significant at the 10% level in specifications (3) and (7)
$^*p < 0.05$; $^{**}p < 0.01$; $^{***}p < 0.001$

### Discussion of results

When we change the context from a game show to a setting with an unspecified asymptomatic disease, we find that traditional risk elicitation measures no longer predict behavior in the task. However, we do not observe a significant treatment effect on our collected metrics of overall protection choices, switching curve intercepts and implied risk aversion.

Our analysis does not provide compelling evidence for a treatment effect as outlined in Claims 8–10 in Sect. 1. We do observe a treatment effect in one dimension: if subjects have low priors, being in the disease treatment causes them to start protection for lower risks. However, no general change in behavior is identifiable. Without more detailed data, we cannot decisively reject our claims.

On interpretation of our findings imply is that different risk attitudes are employed in different settings and it takes different risk attitude measures, attuned to context, to predict them. This would be in line with previous work such as Szrek et al. (2012) that shows that risk aversion can be highly context dependent. In particular, traditional measures perform poorly where health is concerned. Crosetto and Filippin (2013a, b) find that while the IG measure does not correlate with risk measures elicited through the German Socio-Economic Panel Study (SOEP) (Wagner et al. 2007) questionnaire and the Domain-Specific Risk Taking Scale (DOSPERT) (Blais and Weber 2006), it does correlate strongly with attitudes to financial investments and gambling, which could explain the significance in regressions using data only from the game show treatment. The BRET also does not correlate with either the SOEP or the DOSPERT, nor does it correlate with attitudes to financial investments and gambling. This might explain why the BRET does not have the expected effects in our experiment.

However, as outlined in the previous section, this is just one interpretation of the results. How could we shed more light on this issue? A within-subject design could be used to see whether subjects indeed change behavior, but learning effects would be likely to bias results. A more health context sensitive measure of risk aversion should be included in the analysis, for example, questions included in the SOEP and the DOSPERT. Inclusion of measures of altruism or general attitudes to health would also provide useful insights. Finally, conducting research in the field would be needed for conclusive evidence.

### Data

The experimental sessions were carried out during the months of February and July of 2015 at the Faculty of Economics, University of Cambridge. We recruited 72 subjects, almost exclusively from the University of Cambridge student population, through the Online Recruitment System for Economic Experiments (ORSEE) (Greiner 2004) with the help of the Judge Business School. Ten 1.5 hour sessions were run with participant numbers ranging from 4 to 10 subjects. A between subject design was used and therefore each subject only took part in one treatment. Treatments varied across sessions only, so the treatment completed by all subjects in one session was identical. 36 subjects completed the game show treatment and 36 subjects completed the disease

**Table 8** Summary statistics

| Variable | Mean | (SD) | Min. | Max. | N |
|---|---|---|---|---|---|
| Male | 0.39 | (0.49) | | | 70 |
| Age | 23.59 | (3.75) | 18 | 35 | 70 |
| % currently in education | 0.96 | (0.2) | No | Yes | 70 |
| Bachelor | 0.5 | (0.5) | No | Yes | 70 |
| Masters | 0.34 | (0.48) | No | Yes | 70 |
| Phd | 0.16 | (0.37) | No | Yes | 70 |

**Table 9** Differences in treatments

| Variable | GS treat. | D treat. | *p* value (t-test) |
|---|---|---|---|
| Male | 0.35 (0.49) | 0.42 (0.5) | 0.591 |
| Age | 23 (3.19) | 24.14 (4.18) | 0.206 |
| Masters | 0.38 (0.49) | 0.31 (0.47) | 0.515 |
| PhD | 0.12 (0.32) | 0.19 (0.4) | 0.385 |
| Science Major | 0.55 (0.09) | 0.5 (0.08) | 0.715 |
| Pence invested in IG (of 200) | 115.41 (58.69) | 104.17 (63.75) | 0.446 |
| Risky choices in MPL (of 10) | 4.38 (1.36) | 3.43 (1.54) | 0.01 |
| Blocks collected in BRET | 42.02 (12.32) | 43.39 (15.07) | 0.682 |
| Fatalism measure (of 4) | 1.18 (1.09) | 0.72 (0.7) | 0.04 |
| % passed quizzes | 0.76 (0.43) | 0.88 (0.33) | 0.241 |
| *N* | 34/32 | 36/35/26 | |

treatment. Unfortunately it became apparent that 2 subjects had not completed the main task but simply clicked through the screens. They were subsequently excluded from the analysis.

Table 8 shows the summary statistics for our remaining sample of 70 observations. 39% of our sample are males and the average age is around 24. 50% of our subjects are studying for their bachelor's degrees and the other 50% for advanced degrees.

With regards to understanding of the main task, from Table 9 there appears to be a qualitative difference across the two treatments. In the game show treatment 24% of subjects fail the quizzes while only 12% do so in the disease treatment.[16]

## Regression tables

See Tables 10 and 11.

[16] Unfortunately, due to a programming error quiz results for one session went unrecorded which means the disease treatment is missing 10 observations.

**Table 10** Regression of switching likelihood of being infected on risk of becoming infected

| | (1)<br>Indiff. likelihood of being infected | (2)<br>Difference in indiff. likelihood |
|---|---|---|
| Risk of becominginfected | 0.546*** | − 0.067*** |
| | (0.021) | (0.016) |
| MPL | − 19.969 | − 1.875 |
| | (11.381) | (8.711) |
| IG | 1.306*** | 0.063 |
| | (0.131) | (0.100) |
| Fatalism | − 16.313 | − 0.938 |
| | (8.670) | (6.637) |
| Sex | − 203.375*** | − 10.000 |
| | (37.105) | (28.402) |
| Masters | 12.125 | 0.312 |
| | (33.338) | (25.519) |
| Phd | − 157.219** | − 10.625 |
| | (55.671) | (42.614) |
| Disease treatment | − 26.906 | − 2.813 |
| | (23.716) | (18.153) |
| _cons | 73.168 | 14.509 |
| | (82.094) | (62.840) |
| $N$ | 1056 | 1056 |
| $R^2$ | 0.633 | 0.029 |

Standard errors in parentheses. Each regression also includes session and subject dummies
$^*p < 0.05$; $^{**}p < 0.01$; $^{***}p < 0.001$

**Table 11** Risk aversion measure in placebo regressions for the *Lower* and *Upper Intercept*

| | (1)<br>LI in GS and D treatment | (2)<br>LI in HIV treatment | (3)<br>UI in GS and D treatment | (4)<br>UI in HIV treatment |
|---|---|---|---|---|
| Protection offered in AET | − 0.524 | − 0.638 | 1.285 | 0.203 |
| | (0.437) | (0.623) | (1.216) | (0.506) |
| MPL | 1.093 | 4.408* | − 5.755 | − 3.072* |
| | (1.172) | (1.779) | (3.264) | (1.445) |
| IG | − 0.014 | − 0.128** | 0.131 | 0.106** |
| | (0.025) | (0.039) | (0.070) | (0.032) |
| Fatalism | 3.128* | − 2.866 | 2.827 | 1.655 |
| | (1.556) | (2.310) | (4.333) | (1.877) |

**Table 11** continued

| | (1) LI in GS and D treatment | (2) LI in HIV treatment | (3) UI in GS and D treatment | (4) UI in HIV treatment |
|---|---|---|---|---|
| Male | − 2.229 | 4.571 | 5.336 | − 4.120 |
| | (2.884) | (4.342) | (8.030) | (3.527) |
| | (4.169) | | (11.609) | |
| Disease treatment | − 12.841 | | − 0.542 | |
| | (6.443) | | (17.941) | |
| Freq. of condom use | | − 1.205 | | 3.503 |
| | | (2.492) | | (2.025) |
| Years in higher education | | − 0.984 | | − .275 |
| | | (1.396) | | (1.134) |
| Estimate of transmission factor | | − 0.261*** | | 0.169** |
| | | (0.070) | | (0.056) |
| _cons | 17.754* | 35.438** | 69.810** | 67.056*** |
| | (7.776) | (12.122) | (21.653) | (9.848) |
| $N$ | 67 | 67 | 67 | 67 |
| $R^2$ | 0.355 | 0.466 | 0.236 | 0.399 |

Standard errors in parentheses. Each regression also includes session dummies and dummies for advanced degrees
$^*p < 0.05$; $^{**}p < 0.01$; $^{***}p < 0.001$

## Experiment screen shots

See Figs. 4, 5, 6, 7 and 8.

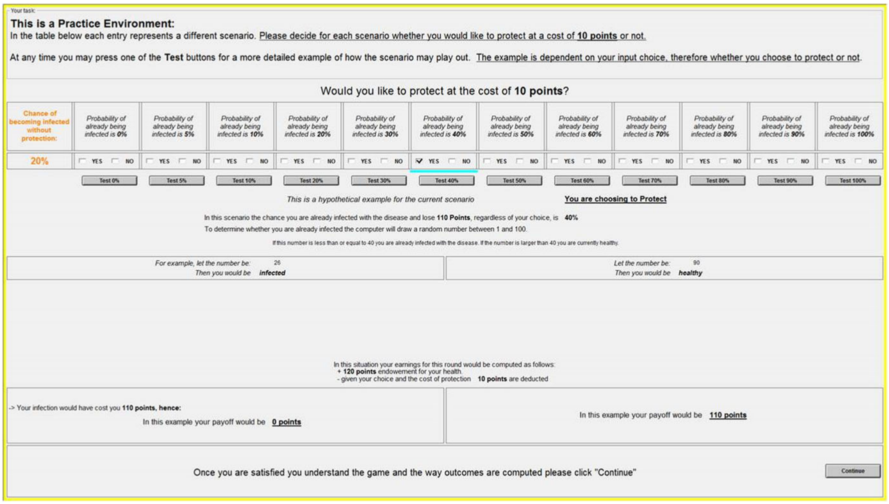

**Fig. 4** Disease treatment: example screen

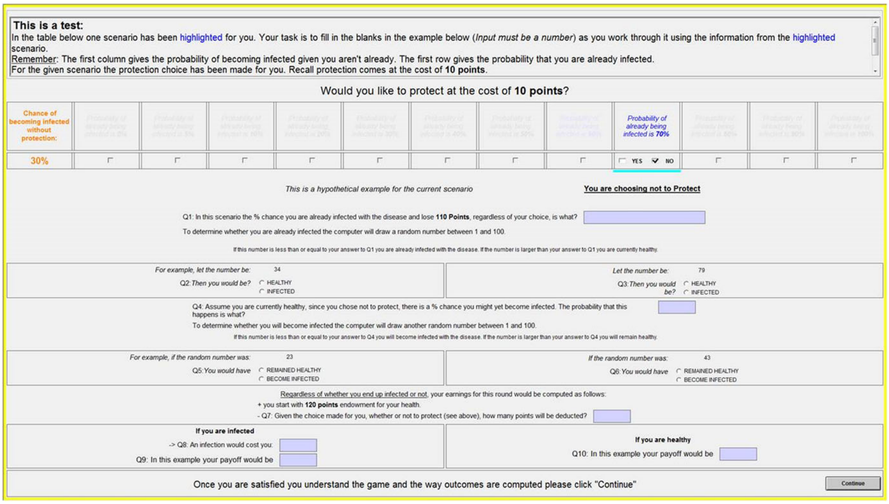

**Fig. 5** Disease treatment: quiz screen

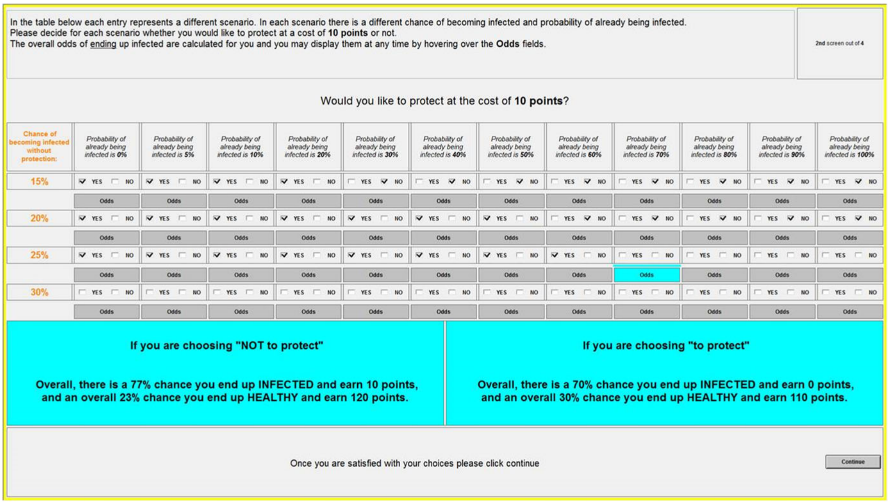

**Fig. 6** Disease treatment: main screen

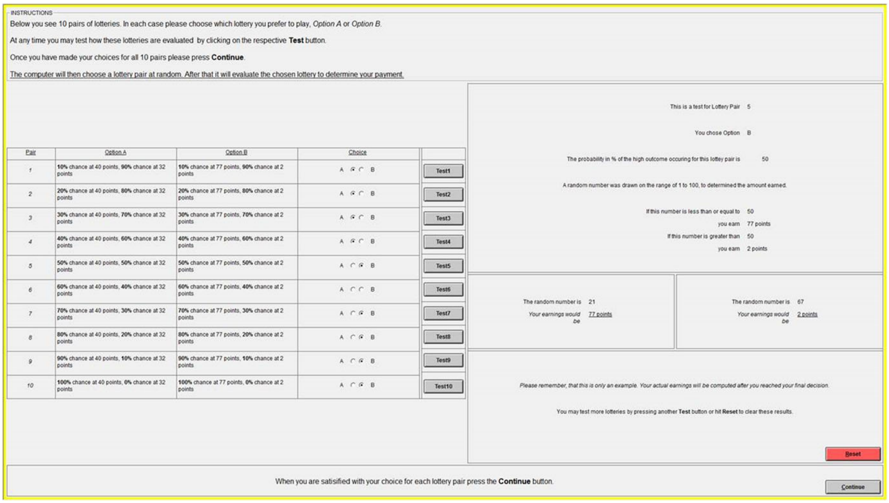

**Fig. 7** Multiple pricelist task: main screen

**Fig. 8** Investment game: main screen
